# Light-activatable manganese carbonate nanocubes elicit robust immunotherapy by amplifying endoplasmic reticulum stress-meditated pyroptotic cell death

**DOI:** 10.1186/s13046-025-03408-5

**Published:** 2025-05-16

**Authors:** Chuan Wu, Mingquan Gao, Weidong Xiao, Xie Huang, Xinrui Yang, Zifei Wu, Xudong Yu, Banghui Mo, Zaizhi Du, Ziqian Shang, Jing Liu, Can Shi, Rong Li, Shenglin Luo, Weidong Wang

**Affiliations:** 1 Department of Radiation Oncology, Sichuan Clinical Research Center for Cancer, Sichuan Cancer Hospital & Institute, Sichuan Cancer Center, School of Medicine, University of Electronic Science and Technology of China, Chengdu, 610054 China; 2 Institute of Combined Injury, State Key Laboratory of Trauma and Chemical Poisoning, Chongqing Engineering Research Center for Nanomedicine, College of Preventive Medicine, Third Military Medical University (Army Medical University), Chongqing, 400038 China; 3 Department of Radiation Oncology, Radiation Oncology Key Laboratory of Sichuan Province, Sichuan Clinical Research Center for Cancer, Sichuan Cancer Hospital & Institute, Sichuan Cancer Center, University of Electronic Science and Technology of China, Chengdu, 610041 China; 4 Department of Pharmacy, Daping Hospital, Third Military Medical University (Army Medical University), Chongqing, 400042 China

**Keywords:** Heptamethine cyanine dye, Phototherapy, Manganese carbonate nanocubes, Image-guided therapy, Photoacoustic imaging

## Abstract

Although tumor immunotherapy has emerged as a promising treatment modality, it faces significant challenges stemming from the immunosuppressive characteristics of the tumor microenvironment (TME), the low immunogenicity of tumors, and the poor specificity of immunoactivation. These factors can hinder the efficacy of immunotherapeutic approaches and lead to immune-related adverse events. This study reports a multifunctional nanocube (Mn-ER-Cy) that integrates Mn carbonate (MnCO_3_) and a photosensitizer (ER-Cy) by targeting tumor-cell endoplasmic reticulum (ER). The results demonstrate that Mn-ER-Cy preferentially accumulates in tumor tissues and is retained within ER organelles, facilitating photothermal therapy (PTT) and photodynamic therapy (PDT) upon exposure to 808 nm light irradiation. Triggered by acidic TME and light irradiation, MnCO_3_ is rapidly degraded to Mn^2+^, which in turn promotes the generation of reactive oxygen species through the Mn^2+^-mimic Fenton reaction, enabling chemical dynamics therapy (CDT). Triple-modal synergistic therapy simultaneously happens in ER to induce excessive ER stress, which subsequently amplify highly immunogenic pyroptotic cell death through activating NLRP3 inflammasome, caspase-1, and gasdermin D (GSDMD) pathway. Meanwhile, the decomposition of MnCO_3_ consumes H^+^ and contributes to an increased intracellular pH by regulating lactic acid levels, thereby counteracting the immunosuppressive acidic TME. Furthermore, Mn-ER-Cy serves as an inherent dual-modality imaging contrast agent for near-infrared fluorescence and photoacoustic imaging, facilitating imaging-guided precision therapy. These findings underscore the potential of Mn-ER-Cy to substantially enhance the efficacy and specificity of tumor immunotherapy, portraying a bright prospect to improve the clinical outcomes of patients with cancer.

**Supplementary Information:**

The online version contains supplementary material available at 10.1186/s13046-025-03408-5.

## Introduction

Malignant tumors remain a significant global health challenge and are the second leading cause of death, accounting for approximately 9.7 million deaths in 2022 [1]. As a promising therapeutic modality, immunotherapy has made substantial progress by activating the patient’s immune system to combat tumors [2]. Strategies such as immune checkpoint inhibitors, chimeric antigen receptor T cell therapy, and tumor vaccines have emerged and shown promising potential in treating various malignancies, substantially improving the prognosis of patients with cancer [3]. Despite some success, immunotherapy still faces tough challenges, including relatively low response rates for limited efficacy, and poor specificity for immune-related adverse events [4, 5, 6]. Therefore, there remains an urgent need to explore more effective and specific strategies for promoting antitumor immune response in a larger population of patients.

The effectiveness of cancer immunotherapy is predominantly limited by the immunosuppressive tumor microenvironment (TME), including the acidic microenvironment and immunosuppressive cells accumulated in the TME, such as myeloid-derived suppressor cells, M2 macrophages, and regulatory T cells (Tregs) [7]. Tumor cells undergo metabolic reprogramming to produce large amounts of lactate, leading to acidification of the TME [8]. This acidic environment not only expedites the proliferation and metastasis of tumor cells but also impairs the function of immune cells [9]. In particular, recent evidence has demonstrated that acidic TME can induce M2 polarization of tumor-associated macrophages (TAMs), impede antigen presentation, and inhibit dendritic cell (DC) activation [10]. Therefore, neutralizing the acidic characteristic of the TME may represent an effective strategy for sensitizing cancer immunotherapy. For example, bicarbonates are a representative class of antitumor adjuvant drugs with good biocompatibility and biodegradability [11]. They are also commonly used as immune modulators to neutralize the acidic TME and improve immunotherapy. Administration of an oral bicarbonate buffer solution combined with anti-PD-1 treatment can effectively inhibit melanoma growth and improve survival rates [12]. However, bicarbonates lack specific targeting of tumor tissues and may alter the pH of healthy tissues, thereby increasing potential side effects. Furthermore, their release and action in vivo are relatively short-lived, making it challenging to sustain the long-term efficacy of treatment. Therefore, enhancing their targeting capability and achieving their controlled release may improve the therapeutic effect.

In addition to the immunosuppressive acidic TME mentioned above, the low immunogenicity of tumors is another critical factor that cannot trigger enough immune response. Traditional treatment modalities, such as chemotherapy and radiotherapy, can induce immunogenic cell death (ICD) via apoptosis, necrosis, and autophagy pathways [13]. However, most of these common strategies are still characterized by limited immune responses. Therefore, shifting the paradigm from low to high ICD would significantly improve the efficacy of immunotherapy and provide better outcomes for cancer patients. Among various programmed cell death mechanisms, pyroptosis has emerged as a promising candidate due to its association with inflammation and the release of danger-associated molecular patterns (DAMPs) that can activate an immune response [14]. Pyroptosis is an inflammatory cell death typically mediated by gasdermin D (GSDMD) proteins, leading to cell lysis and the release of proinflammatory cytokines, which can enhance immune cell recruitment and activation [15]. Despite the potential benefits of inducing pyroptosis in tumors, the underlying mechanisms governing its activation and effects on tumor immunity are still not fully understood. Most importantly, developing specific therapeutic strategies that effectively induce pyroptosis in tumor cells while minimizing potential adverse effects on normal tissues is necessary.

In the past decade, researchers [16, 17, 18, 19] have identified and developed a novel class of heptamethine cyanine (Cy) fluorescent dyes that preferentially accumulate in tumor cells. Mechanism studies have demonstrated that their tumor-specific active intake is mainly mediated by organic anion-transporting polypeptides (OATPs), which are cellular membrane transporters overexpressed on the surface of various cancer cells [20]. In further studies, a series of tumor-targeting photosensitizers have been designed by our group for tumor imaging-guided precise photothermal therapy (PTT)/photodynamic therapy (PDT) based on these active-targeting fluorescent Cy dyes [21, 22, 23, 24]. Among them, a few tumor/endoplasmic reticulum (ER) dual-targeting Cy photosensitizers have induced robust ICD for cancer photoimmunotherapy [25]. The ER is a critical organelle for protein folding, lipid synthesis, and calcium ion (Ca^2+^) storage, which are vital for maintaining cellular homeostasis [26]. Under external stimuli or endogenous metabolic disturbances, endoplasmic reticulum stress (ERS) is a significant protective response crucial for preserving cellular stability and function [27]. However, more and more recent studies have indicated that excessive ERS can lead to pyroptotic cell death and produce high immunogenic tumor antigens through mechanisms involving the activation of nucleotide oligomerization domain-like receptor protein (NLRP) 1 and 3 inflammasome and the disruption of intracellular Ca²⁺ homeostasis and protein synthesis [28, 29, 30].

**Scheme 1:**
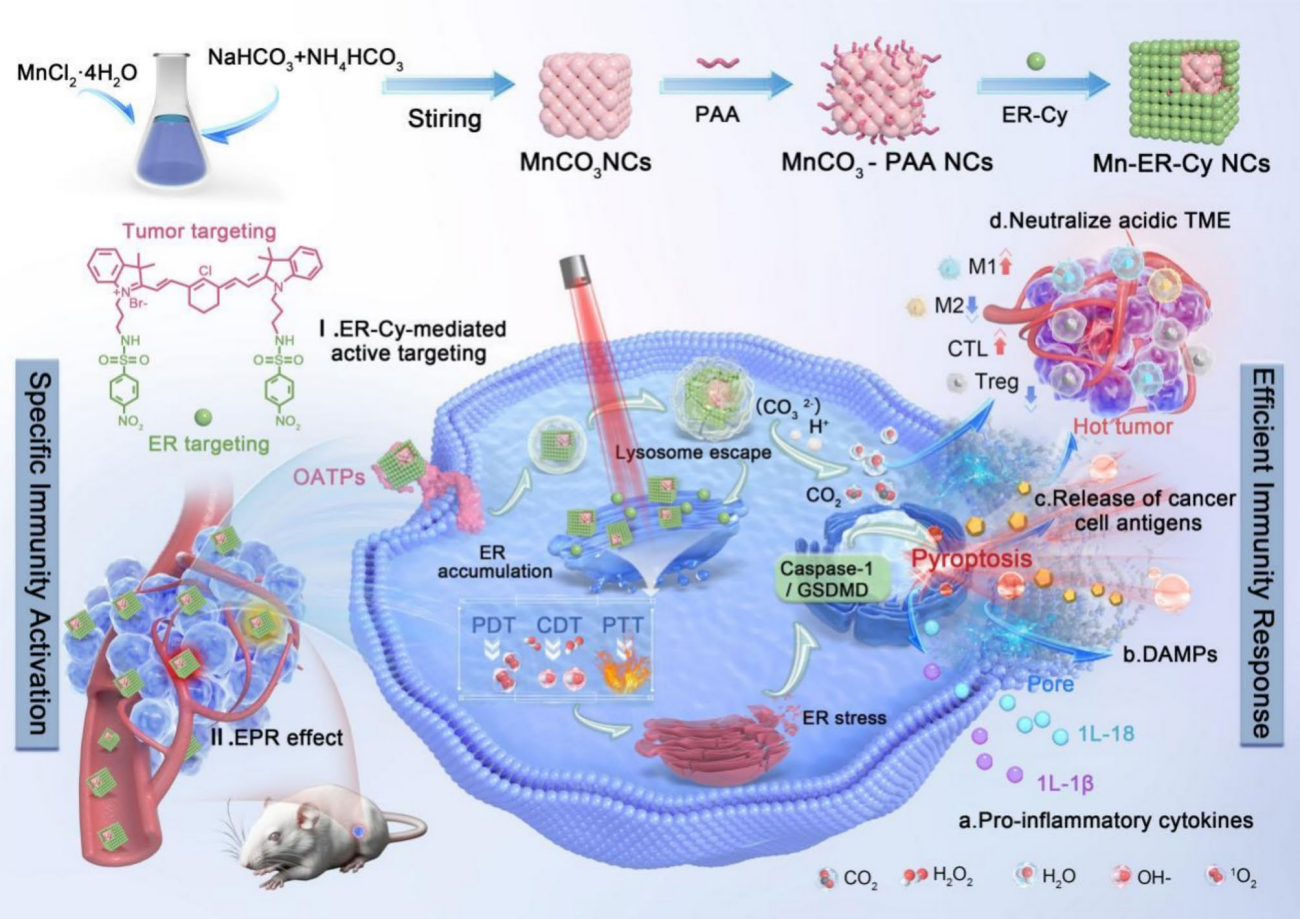
Schematic illustration of the preparation and anti-tumor mechanism of Mn-ER-Cy. Mn-ER-Cy was prepared by the surface modification of MnCO_3_ with PAA and a tumor-targeting photosensitizer ER-Cy. Mn-ER-Cy exhibited a multimodal therapeutic effect of PTT/PDT/CDT, simultaneously happening in the tumor-cell ER. Consequently, excessive ER stress was induced, efficiently boosting pyroptotic cell death, counteracting the immunosuppressive TME, specifically activating and efficiently amplifying antitumor immunity

Based on the abovementioned studies, the current study aimed to construct a multifunctional nanocubes (NCs; designated as manganese-ER-Cy [Mn-ER-Cy]) by integrating Mn carbonate (MnCO_3_) and a tumor/ER dual-targeting Cy photosensitizer (ER-Cy) [25] modified on the surface of MnCO_3_. The Mn-ER-Cy was successfully prepared, characterized, and evaluated via in vitro and in vivo experiments. Our results demonstrate that Mn-ER-Cy can preferentially accumulate in tumor cells and be retained in ER. Under acidic TME and 808 nm light irradiation, Mn-ER-Cy exerts a multimodal antitumor effect, including PTT/PDT facilitated by ER-Cy, chemical dynamics therapy (CDT) facilitated by Mn^2+^-mimic Fenton reaction. Multimodal therapeutic approaches efficiently boost pyroptotic death in tumor cells by inducing excessive ERS, counteracting the immunosuppressive acidic TME, and amplifying antitumor immunity (Scheme 1). This study may offer a promising new paradigm for achieving high efficacy and specificity in tumor immunotherapy.

## Materials and methods

### Chemical reagents

Cetyltrimethylammonium bromide (CTAB), cyclohexane, ammonium bicarbonate (NH_4_HCO_3_), polyacrylic acid (PAA), and sodium carbonate (Na_2_CO_3_) were sourced from Aladdin. n-Pentanol was procured from MACK. Manganese(II) chloride tetrahydrate (MnCl_2_·4 H_2_O) was supplied by Xi’an Qiyue Biotechnology Co., Ltd. Sodium bicarbonate (NaHCO_3_) was obtained from Shanghai Sansheng Biotechnology Co., Ltd., and Tris-HCl buffer was purchased from Sigma Aldrich.

### Synthesis of ER-Cy and MnCO_3_ NCs

The synthesis method for cyanine photosensitizer ER-Cy can be referenced from the experimental procedures detailed in a previously published study by our research group [25].

MnCO_3_ NCs were synthesized using a microemulsion-mediated solvothermal method, based on Hu’s previous report [31] with minor modifications for process optimization. First, prepare the following solutions: Solution A: Dissolve 1.334 g of CTAB in a mixture of 40 mL of cyclohexane and 2 mL of n-pentanol. Solution B: Dissolve 5.336 g of CTAB in a mixture of 160 mL of cyclohexane and 8 mL of n-pentanol. Solution C: Dissolve 6.66 mmol of manganese(II) chloride tetrahydrate in 1.334 mL of deionized water. Solution D: Dissolve 12.66 mmol of sodium bicarbonate and 0.66 mmol of ammonium bicarbonate in 5.336 mL of deionized water. Next, add Solution C to Solution A to form a clear emulsion, and then add the supernatant from Solution D to Solution B to create a slightly translucent emulsion. Stir both emulsions separately at 700 rpm for 3 h, then combine them and stir for an additional hour. Wash the resulting mixture alternately with ethanol and deionized water for three cycles, centrifuging at 10,000 rpm for 10 min after each wash to separate the precipitate. Finally, the precipitate was vacuum dried to obtain MnCO_3_ NCs.

### Preparation of MnCO_3_-PAA and Mn-ER-Cy

The preparation of MnCO_3_-PAA was based on the method reported by Lei Ren et al. [32], with minor modifications. Initially, 0.03 mmol of polyacrylic acid (PAA) was dissolved in 90 mL of deionized water, and the pH of the solution was adjusted to 8.0 using 0.5 M sodium carbonate (Na_2_CO_3_) to form a PAA-Na solution. Subsequently, 5 mL of this PAA-Na solution was added to 160 mL of water containing 40 mg of MnCO_3_. The mixture was stirred at room temperature for 4 h. After stirring, the solution was centrifuged at 10,000 rpm for 10 min, washed with water three times, and then vacuum-dried to obtain MnCO_3_-PAA for further use.

To prepare Mn-ER-Cy, 20 mg of MnCO_3_-PAA was dissolved in 40 mL of Tris-HCl buffer solution at pH 8.5. Next, 2.0 mg of ER-Cy dye was dissolved in 0.5 mL of DMSO and added to the reaction mixture. The mixture was stirred at room temperature at a speed of 700 rpm for 8 h. After stirring, centrifuge was conducted at 10,000 rpm for 5 min. Subsequently, the resulting Mn-ER-Cy was washed three times with deionized water to remove any unbound ER-Cy. Finally, the Mn-ER-Cy was vacuum-dried and stored in a sealed container at 4 °C for further use.

### Mn^2+^ release determination

500 µg/mL of Mn-ER-Cy were dispersed in PBS buffer solution at different pH values (7.4 and 6.5) and then was gently agitated at 37 °C with a rotation speed of 120 rpm. At predetermined time intervals (1 h, 2 h, 6 h, 8 h, 12 h, and 24 h), a 1 mL aliquot was withdrawn, centrifuged at 10,000 rpm for 10 min, and the supernatant was subsequently collected. The concentration of released Mn²⁺ was quantified using inductively coupled plasma mass spectrometry (ICP-MS) with an iCAP TQ ICP-MS/MS system (Thermo Scientific, USA).

### Photothermal evaluation

1.5 mL of aqueous solutions containing Mn-ER-Cy at varying concentrations (0, 12.5, 25, 50, 100 µg/mL) were placed into Eppendorf tubes. The samples were irradiated continuously with an 808 nm NIR laser at a power density of 0.8 W/cm² for 5 min. The temperature changes were recorded every 30 s using a FLUKE NIR thermal imaging camera. Finally, the photothermal conversion efficiency (PCE) of the Mn-ER-Cy was assessed using methodologies established in our previous research [33]. Briefly, the samples containing 50 µg/mL Mn-ER-Cy and ddH_2_O were continuously irradiated with an 808 nm laser (0.8 W/cm²) for 5 min. Temperature changes were monitored every 30 s using a thermal imaging camera during irradiation. After turning off the laser, the cooling process of both Mn-ER-Cy and ddH_2_O solutions was similarly monitored every 30 s until they cooled to room temperature. The PCE (η) of Mn-ER-Cy was calculated using Eq. (1):

1 $$\eta = \frac{{hs\left( {{T_{\max }} - {T_{amb}}} \right) - {Q_{dis}}}}{{I\left( {1 - {{10}^{ - {A_{808}}}}} \right)}}$$

Where the value of hs is determined by Eq. (2), with h representing the heat transfer coefficient and s denoting the surface area of the container. Q_dis_ represents the heat dissipated by the solvent and container from the laser irradiation. I indicates the laser power, and A_808_ is the absorbance of the 50 µg/mL Mn-ER-Cy aqueous solution at 808 nm:

2 $$hs = \frac{{m{C_{solution}}}}{{{\tau _s}}}$$

In Eq. (3), m represents the mass of the sample solution, C stands for the specific heat capacity of the solution, and τ s is the time constant derived from the cooling curve versus time:

3 $${\tau _s} = - \frac{t}{{\ln \theta }}$$

Where t represents time, and θ is the dimensionless driving force parameter calculated by Eq. (4):

4 $$\theta = \frac{{T - {T_{amb}}}}{{{T_{\max }} - {T_{amb}}}}$$

T_max_ denotes the maximum temperature reached, and T_amb_ represents the ambient temperature.

### Degradation characteristics of Mn-ER-Cy

200 µg/mL Mn-ER-Cy was dissolved in aqueous solutions with different pH levels (pH 7.4 and pH 6.5) and placed in a 12-well plate. Using a 100 µL pipette, a small amount of the solution was drawn and then dripped onto a copper grid. After 60 min, a small amount of the liquid was again sampled and processed in the same manner. To demonstrate that laser irradiation can promote the degradation of nanoparticles, an additional experimental group at pH 6.5 combined with laser irradiation was conducted for comparison. Samples were taken and dripped onto the copper grid at both 0 min and 60 min, allowing them to air dry naturally. The degradation of Mn-ER-Cy was observed through Transmission Electron Microscopy (TEM) morphological analysis.

### Singlet oxygen sensor green (SOSG) generation detection

100 µg of SOSG was dissolved in 33 µL of methanol to prepare a 5 mM SOSG stock solution, which was kept on ice for later use. A 50 µg/mL Mn-ER-Cy solution was then prepared in a 2% methanol aqueous solution and added to a 12-well plate. Next, 5 µM SOSG solution was added and mixed thoroughly. After irradiation with an 808 nm laser for 5 min, the solution was immediately transferred to a cuvette for fluorescence emission spectrum analysis using a fluorescence spectrophotometer (excitation wavelength: 504 nm, emission wavelength: 525 nm).

### Cell culture and experimental groups

The 4T-1 mouse breast cancer cells were obtained from the Shanghai Institute of Life Sciences Cell Bank and cultured in DMEM supplemented with 1% penicillin/streptomycin (Gibco) and 10% FBS (Hyclone) at 37 °C in a 5% CO_2_ atmosphere. The experiments were divided into four groups with the following treatments: Control group (I): Cultured in complete DMEM medium. Laser alone group (II): Cultured in complete DMEM medium and exposed to 808-nm NIR laser irradiation (0.8 W/cm² for 5 min). Mn-ER-Cy alone group (III): Cultured in DMEM containing 50 µg/mL Mn-ER-Cy. Mn-ER-Cy (+) group (IV): Incubated with 50 µg/mL Mn-ER-Cy in DMEM for 4 h, followed by exposure to 808 nm NIR laser irradiation (0.8 W/cm² for 5 min).

### Cell internalization analysis

4T-1 cells were seeded at a density of 5 × 10^4 cells per well in 35 mm confocal culture dishes and allowed to adhere. Following adherence, 1 mL of DMEM containing 50 µg/mL Mn-ER-Cy was added and incubated for designated time points (1, 2, 4, 8, 12, and 24 h). After staining the cell nuclei with DAPI, fluorescence intensity changes were assessed using a CLSM to evaluate the uptake of Mn-ER-Cy by the cells at different time points.

### Subcellular localization analysis

4T-1 cells were seeded at a density of 5 × 10^4 cells per well in 35 mm confocal culture dishes and allowed to adhere. After adherence, 1 mL of DMEM containing 50 µg/mL Mn-ER-Cy was added, and the cells were incubated for designated time points (1, 2, 4, and 8 h). Subsequently, the cells were incubated with 1 mL of Lyso-Tracker Green (cat#: C1047S, Beyotime) and ER-Tracker Green probes (cat#: C1042M, Beyotime) at 37 °C for 20 min. Images were captured using a CLSM, and the colocalization of Mn-ER-Cy with lysosomes and the ER was analyzed using ImageJ software.

### Reactive oxygen species (ROS) generation determination

4T-1 cells were seeded in a 35 mm culture dish and treated according to the designated experimental groups, followed by an additional 24-hour incubation period. DCFH-DA was diluted 1:1000 in serum-free medium to a final concentration of 10 µmol/L, and 1 mL of the solution was added to the cells for a 20-minute incubation at 37 °C. Upon completion of the incubation, the cells were washed with serum-free culture medium to remove any unincorporated dye. Subsequently, intracellular ROS levels were visualized and documented using a fluorescence microscope (1 × 51, OLYMPUS, Japan).

### In vitro and in vivo PA imaging

To investigate the PA imaging capability of Mn-ER-Cy, various concentrations (0, 12.5, 25, 50, 100, and 200 µg/mL) of Mn-ER-Cy were prepared in aqueous solution. These solutions were then transferred into test tubes for ex vivo imaging. The tubes were placed in the near-infrared PA imaging system (LOIS-3D, TomoWave Laboratories, USA) and imaged using 808 nm near-infrared laser excitation.

For in vivo NIR PA imaging, tumor-bearing nude mice were positioned on the imaging system platform prior to drug administration. Baseline PA images were acquired using 808 nm NIR laser excitation. Following this, Mn-ER-Cy was administered intravenously at a dosage of 20 mg/kg. PA imaging was conducted at predetermined time points (0, 2, 6, 12, and 24 h post-injection). The PA signal intensities within the tumor regions were semi-quantitatively analyzed over these time intervals to assess the accumulation of Mn-ER-Cy in the tumor tissues.

### In vivo NIR fluorescence imaging

The AniView multi-modal in vivo imaging system (AV100-0923-8304D, BLTPhotoTech) was utilized to continuously monitor the biodistribution and tumor-targeting characteristics of Mn-ER-Cy. Briefly, Mn-ER-Cy was administered intravenously to 4T-1 tumor-bearing BALB/c mice. Subsequently, in vivo near-infrared (NIR) imaging was conducted at various time intervals following the injection. At predetermined time points, the mice were euthanized by cervical dislocation, and major organs (including the heart, liver, spleen, lungs, kidneys, muscle, and small intestine), as well as tumor tissues, were collected for ex vivo NIR imaging. The fluorescence intensity in the tumor region and other organs was semi-quantitatively analyzed using AniView software.

### Extracellular and intracellular pH detection

For the assessment of extracellular pH, 4T-1 cells were seeded at a concentration of 1 × 10^5 cells/mL in a 6-well plate and treated according to the designated experimental groups. A blank control group consisting of fresh medium without cells was also established. All plates were incubated in a 37 °C, 5% CO_2_ incubator. At predetermined time points (4 h, 12 h, and 24 h), the pH of the cell culture supernatants from each group was measured using a laboratory pH meter (PHS-3 C, Leici, China).

For intracellular pH measurement, 4T-1 cells were seeded in a 35 mm culture dish and treated according to the experimental groups, followed by a 24-hour incubation period. Intracellular pH was assessed using a cell pH detection kit. Specifically, 1 mL of BBcellProbe^®^ P01 staining working solution (cat#:BB-481211, BestBio) was added to the treated cells and incubated at 37 °C for 60 min. The cells were then washed three times with PBS, and 1 mL of serum-free culture medium was added. Finally, CLSM was performed immediately, utilizing an excitation wavelength of 488–506 nm and an emission wavelength of 526–536 nm.

### Transcriptome sequencing

The 4T-1 cells were treated according to their assigned groups, specifically the Control group and the Mn-ER-Cy with laser group. After a 24-hour treatment period, the cells were collected and adjusted to a density of 2 × 10^7 cells/mL. The samples were then frozen in liquid nitrogen for 5 min and subsequently stored at -80 °C. Transcriptome sequencing experiments and analyses were conducted by Shanghai Bohan Biotechnology Co., Ltd.

### NLRP3 inhibitor and _Si_RNA transfection

4T-1 cells were pre-treated with 50 nM of the NLRP3 inhibitor MCC950 (catalog #: HY-12815, MCE) for 6 h or transfected with NLRP3 _si_RNAs (GenePharma) for 48 h. Following this, the cells were incubated with 50 µg/mL of Mn-ER-Cy for 4 h, after which they were exposed to 808 nm laser irradiation (0.8 W/cm², 5 min). After 24 h, the morphology of cell pyroptosis and the expression of related proteins were assessed. The sequences of NLRP3 _si_RNAs are included in Table S1.

### In vivo antitumor therapy

All animal studies conducted in this research received the necessary approval from the Ethics Committee of the Third Military Medical University in Chongqing, China (approval number: AMU2020067). The study adhered rigorously to the ethical guidelines established by the National Institutes of Health for conducting animal experiments. Female BALB/c mice, aged six weeks and weighing between 18 and 22 g, were sourced from the Laboratory Animal Center at the Third Military Medical University. Following a week of acclimatization, each mouse received a subcutaneous injection of 100 µL of PBS containing 2 × 10^6 4T-1 cells in the right flank. Once the tumors had reached an approximate volume of 100 mm³, the tumor-bearing mice were randomly allocated into four treatment groups (*n* = 5 per group) as follows: Group I received a 100 µL PBS control via intraperitoneal injection; Group II was subjected to 808-nm laser irradiation at a power density of 0.5 W/cm² for 5 min; Group III was administered Mn-ER-Cy treatment at a dosage of 20 mg/kg via intraperitoneal injection on days 1 and 2; and Group IV underwent 808 nm laser irradiation 24 h post-Mn-ER-Cy injection. Throughout the experiment, the body weight and tumor volume of the mice were monitored every three days using an electronic balance and caliper, respectively. On day 21, all mice were humanely euthanized, and their tumor tissues were subsequently harvested, weighed, and photographed. The harvested tumor tissues were subsequently fixed in 4% paraformaldehyde for immunohistochemistry (IHC), hematoxylin and eosin (H&E) staining, and TdT-mediated dUTP nick end labeling (TUNEL) analysis.

### Immune response analysis

For dendritic cell (DC) maturation detection, tumor-draining lymph nodes were isolated from tumor-bearing mice in each experimental group three days post-treatment. These lymph nodes were processed to generate a single-cell suspension by mechanically disrupting them using a 70-µm cell strainer. The resulting cell solution was then resuspended in PBS to achieve a cell density of 1 × 10^7 cells/mL. Subsequently, the cells were incubated with antibodies against lineage markers: APC-Cy7 anti-mouse FVS780 (Cat#:565388, BD Biosciences), PE anti-mouse CD11c (Cat#:12-0114-82, eBioscience), APC anti-mouse CD80 (Cat#: 17-0801-82, eBioscience), and PE-Cy7 anti-mouse CD86 (Cat#:25-0862-82, eBioscience), following the manufacturer’s instructions.

For the infiltration analysis of cytotoxic T lymphocytes (CTLs) and regulatory T cells (Tregs), tumor-bearing mice from each experimental group were euthanized on the sixth day post-treatment, and tumors were collected. The tumors were dissected into small fragments, homogenized, and filtered through a 70-µm cell sieve to obtain a single-cell suspension. This cell suspension was then incubated with antibodies against FITC anti-mouse CD3 (Cat#:11-0031-81, eBioscience), PE-Cy7 anti-mouse CD4 (Cat#:25-0041-82, eBioscience), PerCP Cy5.5 anti-mouse CD8 (Cat#:45-0081-82, eBioscience), PE anti-mouse FoxP3 (Cat#:320008, BioLegend), and BV421 anti-mouse CD25 (Cat#:102034, BioLegend), according to the recommended protocols provided by the manufacturers. Following antibody incubation, the samples were analyzed using flow cytometry, which facilitated the quantification of the percentage of positive cell populations with the assistance of FlowJo Flow Cytometric Analysis Software.

### Bilateral tumor model

4T-1 tumor-bearing BALB/c female mice, with tumors approximately 100 mm³ in size located on the left flank (proximal tumor), were randomly assigned to four groups (*n* = 5 per group) and received the desired treatment as previously described. On day 3, 4T-1 cells (3 × 10^5 cells per mouse) were inoculated into the right flank of the BALB/c mice to establish a distant tumor. Throughout the experiment, the body weight and tumor volume of the mice were monitored every three days using an electronic balance and caliper, respectively. On day 21, all mice were humanely euthanized via inhalation anesthetics, after which their tumor tissues were harvested, weighed, and photographed.

### Lung metastasis model

For the in vivo anti-metastatic experiment, 4T-1 tumor-bearing BALB/c female mice were randomly assigned to four treatment groups (*n* = 3 per group) and received the previously described treatment regimen. On day 3 post-treatment, 100 µL of PBS containing 1 × 10^6 4T-1 cells was injected into the tail veins to induce lung metastasis. On day 21, all mice were sacrificed, and their lungs were surgically excised for imaging and H&E staining.

### Survival monitoring

To monitor the survival rate of tumor-bearing mice, animals were treated as previously described following tumor inoculation (*n* = 10 per group). The survival status of each group was recorded every three days. Mice were classified as deceased when their tumor volume reached 2000 mm³ or when ulceration occurred. The total observation period was 45 days.

### Statistical analysis

Data were processed, analyzed, and plotted using Origin (Version 2019b, Northampton, MA, USA) and GraphPad Prism (Version 9.0, La Jolla, CA, USA). Results are expressed as mean ± standard deviation (SD). For data meeting the assumptions of normal distribution and homogeneity of variance, one-way analysis of variance (ANOVA) was used to assess the significance of differences between group means. If ANOVA indicated statistical significance, post hoc analysis using the least significant difference (LSD) test was performed for group comparisons. A *p*-value less than 0.05 was considered statistically significant.

## Results and discussion

### Preparation and characterization of Mn-ER-Cy NCs

**Fig. 1:**
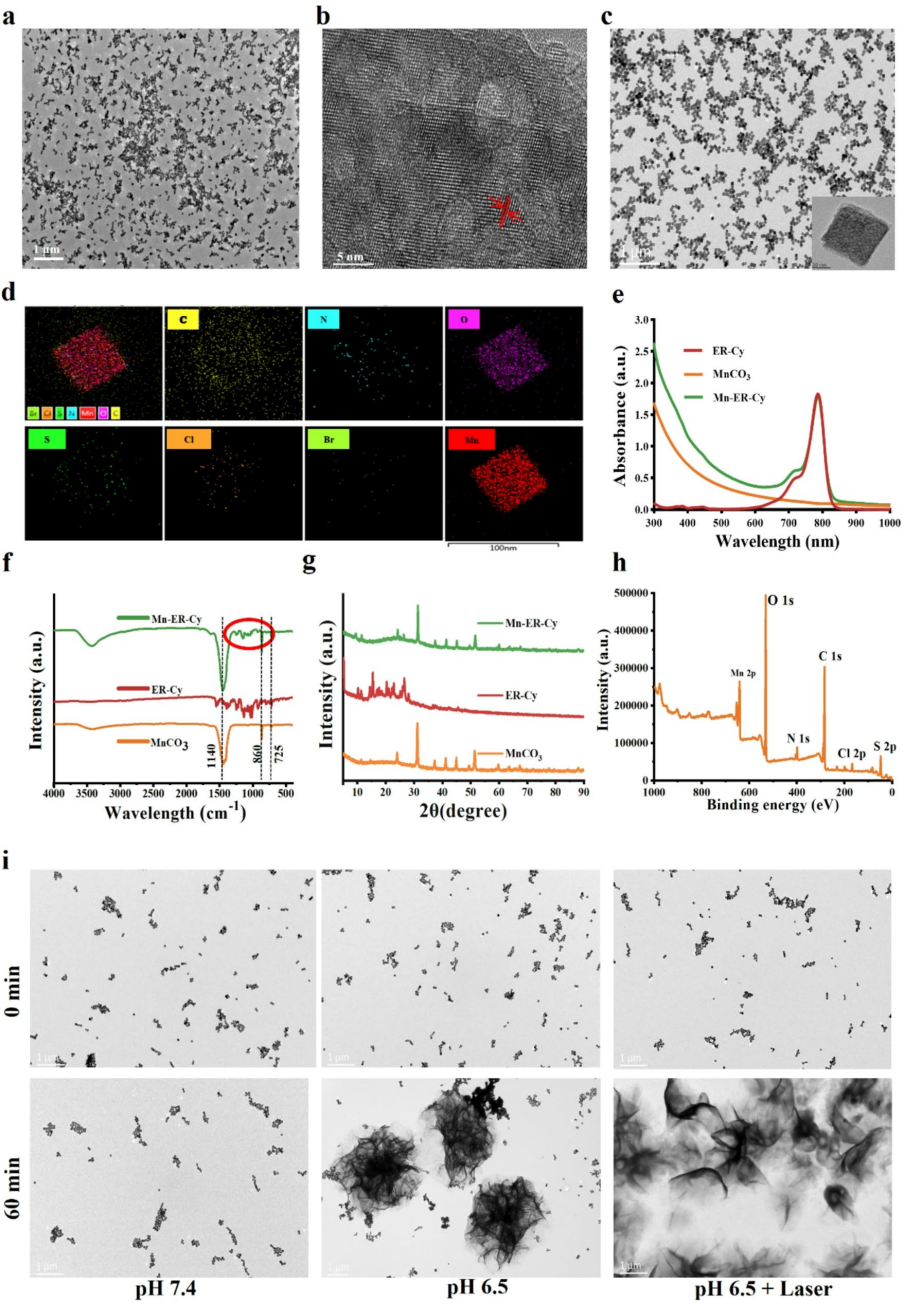
Synthesis and characterization of Mn-ER-Cy. (**a**) Representative TEM image of MnCO_3_. (**b**) Representative high-resolution TEM image of MnCO_3_. (**c**) Representative TEM image of Mn-ER-Cy. (**d**) Elemental mapping analysis showing the distribution of carbon (C), nitrogen (N), oxygen (O), sulfur (S), chlorine (Cl), bromine (Br), and manganese (Mn) in Mn-ER-Cy. (**e**) UV-vis-NIR absorption spectrum of MnCO_3,_ ER-Cy, and Mn-ER-Cy in phosphate-buffered saline (PBS). (**f**-**h**) FTIR spectrum, XRD pattern, and XPS spectrum of Mn-ER-Cy. (**i**) TEM observation of Mn-ER-Cy degradation following exposure to acidic conditions or laser irradiation. Scale bar = 1 μm

MnCO_3_ NCs were synthesized using a microemulsion-mediated solvothermal method [31]. Transmission electron microscopy (TEM) images demonstrated that MnCO_3_ exhibited a cubic morphology (Fig. 1a). High-angle annular dark-field scanning TEM images clearly showed the lattice fringes of MnCO_3_ (Fig. 1b), with an observed lattice spacing of 0.278 nm corresponding to the (104) diffraction plane [34]. Second, polyacrylic acid (PAA) bound to the MnCO_3_ surface, forming MnCO_3_–PAA owing to the strong affinity between the carboxyl groups and metal ions [32]. Finally, ER-Cy was chemically synthesized based on our previously reported protocols [25] and assembled onto the MnCO_3_-PAA surface through electrostatic and hydrophobic interactions, followed by self-polymerization to produce Mn-ER-Cy (Fig. 1c). Energy-dispersive X-ray spectroscopy element mapping demonstrated a homogeneous distribution of carbon (C), nitrogen (N), oxygen (O), sulfur (S), chlorine (Cl), bromine (Br), and manganese (Mn) within Mn-ER-Cy (Fig. 1d). The hydrodynamic diameter of Mn-ER-Cy (∼ 108 nm) was larger than that of MnCO_3_ (∼ 86 nm) (Fig. S1a). The zeta potential significantly shifted from 10.5 ± 8.65 to 43.1 ± 8.29 mV (Fig. S1b). UV-Vis–near-infrared (NIR) absorption and fluorescence emission spectra showed a characteristic absorption peak at 786 nm and a fluorescence emission wavelength of ER-Cy of 825 nm (Fig. 1e and Fig. S2). The Fourier transform infrared (FTIR) spectrum of Mn-ER-Cy not only displayed characteristic absorption peaks centered at 725, 860, and 1442 cm^− 1^ corresponding to MnCO_3_ but also revealed peaks associated with the functional groups of ER-Cy (Fig. 1f). Crystalline structures were characterized by X-ray diffraction (XRD) (Fig. 1g), and all characteristic peaks were consistent with that of the standard MnCO_3_ powder (JCPDS No. 44–1472) [34]. XPS analysis revealed distinct absorption peaks for S and Cl elements in the spectrum, indicating the successful coating of ER-Cy onto the MnCO_3_ surface (Fig. 1h).

MnCO_3_ is stable under neutral and alkaline pH environments, whereas it can decompose into Mn^2+^ and CO_2_ under acidic buffer conditions [35]. After 8 months of storage at − 20 °C, no discernible changes in morphology or particle size were observed, indicating excellent long-term stability. In addition, the hydrodynamic diameter, polydispersity index (PDI), and UV-Vis-NIR absorption profile of Mn-ER-Cy did not exhibit significant changes after 21 days of storage in PBS solutions under pH 7.4 (Fig. S3a-d). Its stability was also assessed using TEM. However, at pH 6.5, Mn-ER-Cy exhibited a time-dependent gradual degradation. Notably, TEM analysis demonstrated a sharp degradation of Mn-ER-Cy after irradiation with 808 nm laser under pH 6.5 (Fig. 1i). Inductively coupled plasma mass spectrometry analysis further substantiated that a weakly acidic environment can obviously enhance Mn^2+^ release, as evidenced by the approximately five times higher concentration of Mn^2+^ at pH 6.5 than at pH 7.4 (Fig. S3e). These findings suggested that Mn-ER-Cy possesses acidity-responsive and light-controllable release properties.

### Cell uptake and ER-targeting accumulation

The cell uptake behaviors of Mn-ER-Cy were investigated in 4T-1 mouse breast cancer cells using confocal laser scanning microscopy (CLSM) imaging. The results revealed that the cell uptake of Mn-ER-Cy increased with incubation time, rapidly reaching saturation at 4 h (Fig. S4). Previous studies have demonstrated that the tumor-specific uptake of heptamethine Cy dyes is actively mediated by OATPs, which are abundantly located on the cellular membrane of various cancer cells, including breast cancer cells [16, 36]. Considering that ER-Cy contains the tumor-targeting heptamethine indocyanine moiety, a large number of Mn-ER-Cy can be actively transported into tumor cells via OATPs. Nevertheless, Mn-ER-Cy is a nanomaterial that could be absorbed by cells partially through the endocytic pathway. Acidic-labile nanomaterials often degrade easily in lysosomes after being endocytosed. To investigate this, the cell uptake process of Mn-ER-Cy was tracked via CLSM imaging and fluorescence colocalization between Mn-ER-Cy and a commercial lysosomal probe (Lyso-Tracker Green), including Pearson colocalization analyses. The colocalization coefficient of Mn-ER-Cy with lysosomes declined over time, decreasing from 0.76 at 4 h to 0.69 at 8 h (Fig. S5). The results indicated that Mn-ER-Cy can escape from lysosomes after being endocytosed. Several methods have been developed to enhance the lysosomal escape of nanomaterials. For instance, cationic substances can act as drug carriers by entering the lysosome and destabilizing the negatively charged lysosomal membrane through electrostatic interactions, thus facilitating lysosomal escape [37]. Another approach involves using materials rich in specific functional groups, such as polyethyleneimine, which can induce lysosomal rupture via a “proton sponge” effect [38]. In this study, it is speculated that Mn-ER-Cy achieve lysosomal escape through the following two mechanisms. First, the cationic nature of Mn-ER-Cy may disrupt lysosomes through electrostatic interactions. Second, Mn-ER-Cy may decompose to release Mn^2+^, which can undergo a Fenton-like reaction with hydrogen peroxide (H_2_O_2_) in lysosomes, generating hydroxyl radicals (·OH). Due to the lack of antioxidant enzymes in lysosomes, hydroxyl radicals may attack lysosomes, specifically targeting membrane lipids and proteins. This damage may cause lysosomal membrane rupture, resulting in altered membrane permeability and facilitating lysosomal escape.

The benzene sulfonamide group can bind to sulfonylurea receptors on the ER membrane, enabling benzene sulfonamide-modified molecules or nanomaterials to accumulate in ER [39, 40]. Considering that the tumor-targeting ER-Cy contains two benzene sulfonamide groups, this study further investigated whether Mn-ER-Cy could accumulate in the ER using a commercial ER tracker for fluorescence colocalization. Results demonstrated that Mn-ER-Cy indeed accumulated in the ER after 4 h, and the Pearson colocalization coefficient of Mn-ER-Cy with an ER tracker reached 0.92 (Fig. S6), suggesting that Mn-ER-Cy rapidly accumulated in the ER after escaping from the lysosomes.

### In vitro multimodal antitumor effect of Mn-ER-Cy

**Fig. 2:**
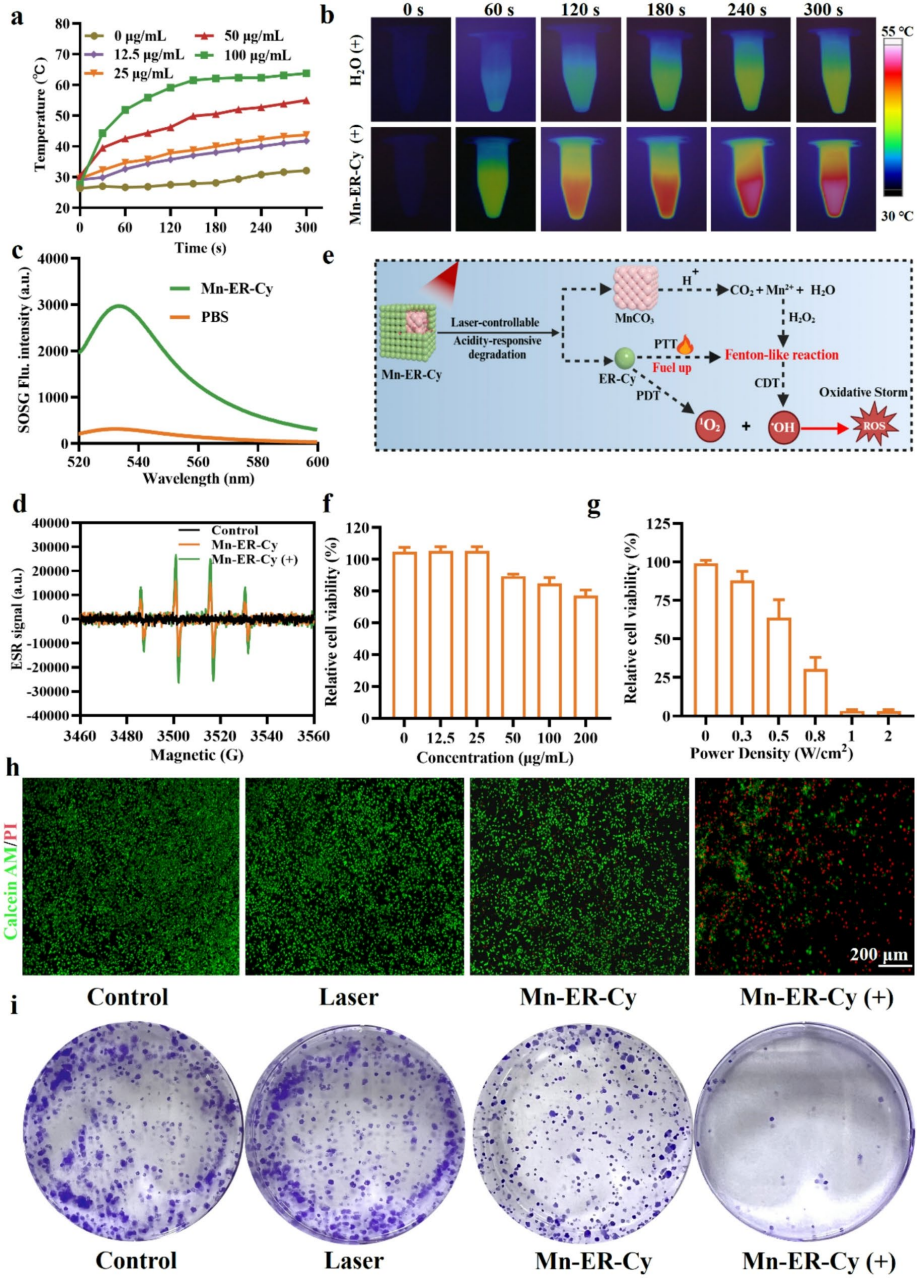
In vitro multimodal antitumor effect of Mn-ER-Cy. (**a**) Temperature profiles, (**b**) infrared thermal imaging, and (**c**) generation of singlet oxygen (¹O_2_) of Mn-ER-Cy upon exposure to 808 nm laser irradiation (0.8 W/cm^2^) for 5 min. (**d**) EPR spectrum illustrating the generation of hydroxyl radicals (·OH) from Mn-ER-Cy with and without laser irradiation. (**e**) Schematic diagram depicting the integrated model of CDT, PTT, and PDT for Mn-ER-Cy. (**f**) Viability of 4T-1 cells after 24-h incubation with varying concentrations of Mn-ER-Cy (*n* = 3, mean ± s.d.). (**g**) Viability of 4T-1 cells after exposure to Mn-ER-Cy (50 µg/mL) combined with 808 nm laser irradiation (*n* = 3, mean ± s.d.). (**h**) Calcein-AM/propidium iodide images illustrating Mn-ER-Cy phototoxicity in 4T-1 cells. Scale bar = 200 μm. Green represents live cells, and red indicates dead cells. (**i**) Clonogenic assays evaluating the effect of Mn-ER-Cy on the proliferation of 4T-1 cells

First, this study investigated the phototherapeutic effect of Mn-ER-Cy under 808 nm laser irradiation because ER-Cy acts as a photosensitizer. Its PTT effect increased with increasing drug concentration (Fig. 2a). As shown in Fig. 2b, the temperature of the control group (PBS) increased from 26.3 °C to 32.1 °C (ΔT = 5.8 °C) after 5 min of laser irradiation. In contrast, the temperature of the Mn-ER-Cy aqueous solution increased immediately after laser irradiation, rapidly reaching 42.5 °C (ΔT = 12.2 °C) within 1 min and increasing to 55 °C (ΔT = 24.7 °C) after 5 min. Photothermal conversion efficiency (PCE), a key indicator for evaluating the performance of photothermal agents (PTAs), is their ability to effectively convert light into thermal energy. Surprisingly, results showed that the PCE of Mn-ER-Cy reached 51.6% (Fig. S7), indicating their effectiveness in converting light into thermal energy. In contrast, many currently reported PTAs, both inorganic and organic, display PCEs of < 40% [41, 42, 43, 44]. The PDT effect of Mn-ER-Cy was also evaluated using a SOSG probe. As shown in Fig. 2c, after irradiation with the 808 nm laser, the fluorescence intensity of SOSG in the Mn-ER-Cy group was nine fold greater than that of the control group. MnCO_3_ NPs showed no significant PDT or PTT effects under 808 nm laser irradiation (Fig. S8), while Mn-ER-Cy exhibited strong PTT and PDT effects under a single excitation wavelength.

Second, previous studies have demonstrated that Mn^2+^ exerts the CDT effect in H_2_O_2_ by promoting ·OH generate through Fenton-like reaction [45]. To verify the CDT effect of Mn-ER-Cy, electron paramagnetic resonance (EPR) was tested for the in situ detection of ·OH. Results demonstrated a significant EPR signal exclusively in the presence of Mn-ER-Cy and H_2_O_2_, confirming its CDT effect (Fig. 2d). Studies showed that PTT/PDT can enhance the catalytic efficiency of the Fenton reaction using Fe^2+^ [46, 47]. Inspired by this, this study further assessed whether the phototherapy effect of Mn-ER-Cy could enhance the Mn^2+^-mimic Fenton reaction, increasing ·OH generation. Results showed that ·OH generated much more after laser irradiation than Mn-ER-Cy alone, indicating that the phototherapeutic effect intensifies the CDT of Mn-ER-Cy induced by the Mn^2+^-mimic Fenton reaction process (Fig. 2e).

Third, Mn-ER-Cy cytotoxicity was evaluated using the CCK-8 assay and flow cytometry. The results show that Mn-ER-Cy did not exhibit obvious cytotoxicity on all kinds of cells when its concentration was below 50 µg/mL. More importantly, Mn-ER-Cy exhibited specific toxicity towards 4T-1 tumor cells rather than other normal cells (MCF-10 A, HUVECs, and RLE-6TN) when the drug concentration reached to 50 µg/mL (15%) (Fig. 2f and Fig. S9). This may be related to the tumor-preferential accumulation of Mn-ER-Cy and the elevated H_2_O_2_ level in the 4T-1 cells, which may trigger a Fenton reaction with Mn²⁺to generate ROS. Encouraged by excellent PTT/PDT/CDT effects, this study further evaluated the antitumor effect of Mn-ER-Cy on 4T-1 tumor cells combined with laser irradiation (+). Cell viability from the CCK-8 assay and live/dead staining demonstrated that Mn-ER-Cy exhibited 15% cell death compared with the control group, likely due to the CDT effect mediated by Mn-ER-Cy. In contrast, the Mn-ER-Cy(+) group demonstrated a significantly higher cell death rate, exceeding 80%, highlighting its exceptional phototherapeutic effect (Fig. 2g-h, and Fig. S10a). Intracellular ROS and ·OH levels were detected using fluorescent probes (Fig. S10b). Results indicated that the Mn-ER-Cy(+) group exhibited the strongest green fluorescence signal, likely due to the combined effects of CDT and PDT (Fig. S11 and Fig. S12). Excessive ROS production can damage the structure and function of the mitochondria, resulting in decreased mitochondrial membrane potential (MMP). The control group exhibited red fluorescence (JC-1 aggregates). In contrast, most cells in the Mn-ER-Cy(+) group displayed green fluorescence (JC-1 monomers), suggesting MMP loss (Fig. S13). The antitumor effect of Mn-ER-Cy was further investigated by inhibiting colony formation (Fig. 2i), migration (Fig. S14), and invasion (Fig. S15). Results indicated that Mn-ER-Cy had a moderate inhibitory effect compared with the control group, whereas the Mn-ER-Cy(+) group exhibited the most significant inhibition. These results revealed that Mn-ER-Cy had a potent antitumor effect by combining CDT, PDT, and PTT to produce significant amounts of ROS and heat.

### Light-activatable Mn-ER-Cy induced ERS and pyroptotic cell death

**Fig. 3:**
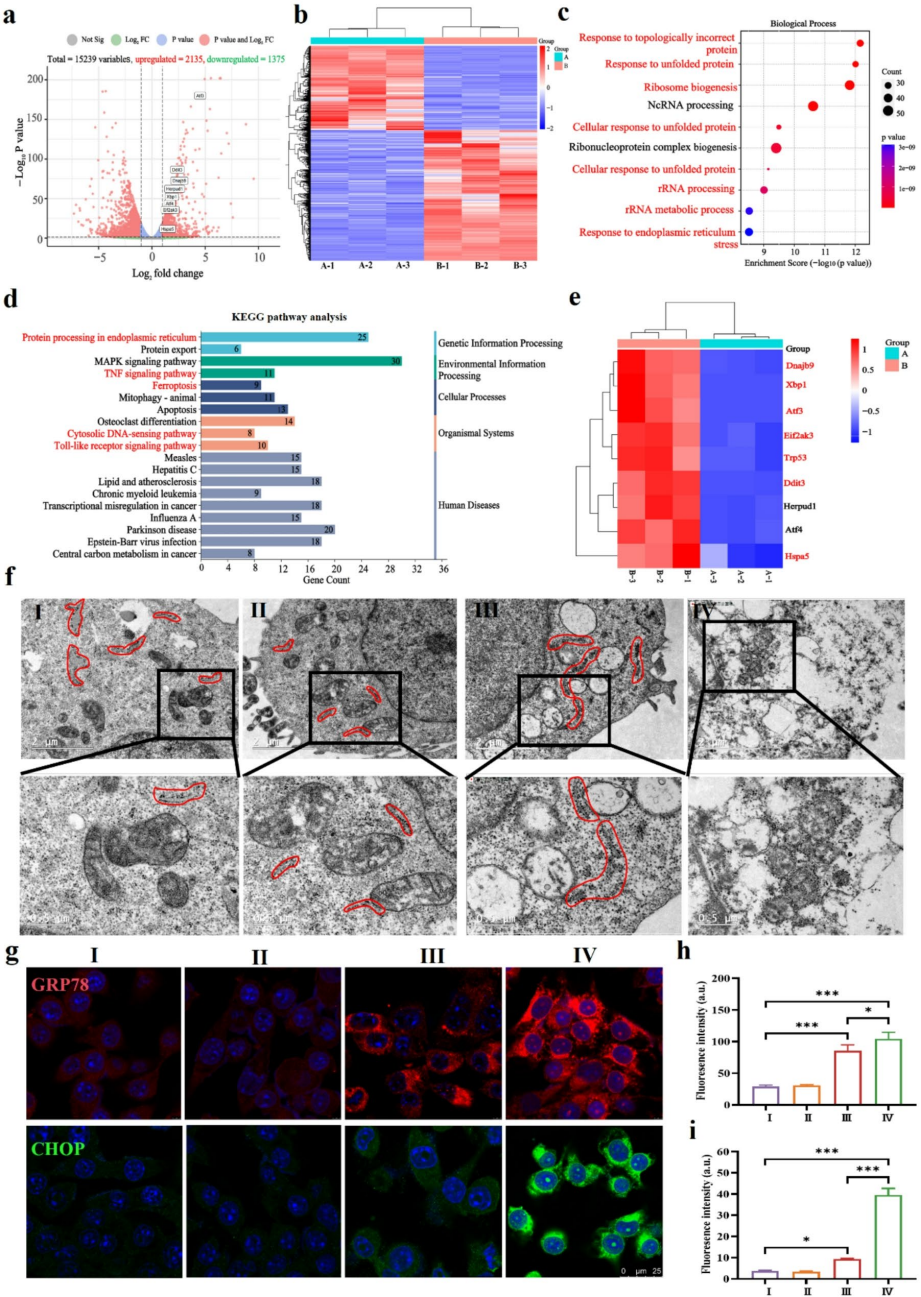
Mn-ER-Cy plus laser irradiation (+) induced excessive ERS. (**a**) Volcano plot displaying DEGs between the Mn-ER-Cy(+) and control groups. (**b**) Heatmap of DEGs, with red indicating high expression and blue indicating low expression levels. A represents the control group, whereas B represents Mn-ER-Cy plus laser irradiation group. (**c**) GO enrichment analysis of upregulated DEGs. (**d**) Scatter plot of the results of KEGG enrichment analysis. (**e**) Heatmap of DEGs involved in the response to ERS. (**f**) Bio-TEM observations of ER morphology changes in 4T-1 cells in each group. (**g**) CLSM images showing CHOP and GRP78 expression levels in 4T-1 cells after various treatments for 24 h. (**h**) and (**i**) Quantitative statistical chart illustrating the fluorescence intensities of CHOP and GRP78 (*n* = 3, mean ± s.d.). The experimental groups were categorized as follows: (I) PBS, (II) 808 nm laser (0.8 W/cm^2^, 5 min), (III) Mn-ER-Cy, and (IV) Mn-ER-Cy with 808 nm laser. Statistical analyses were conducted using one-way ANOVA followed by Tukey’s test. *p < 0.05, ***p < 0.001

The molecular mechanisms of cell death induced by Mn-ER-Cy(+) were next explored using RNA transcriptome sequencing. The volcano plot revealed that there were 3510 differentially expressed genes (DEGs) in the Mn-ER-Cy(+) group, consisting of 2135 upregulated and 1375 downregulated genes compared with the control group (Fig. 3a). To further illustrate the consistency of gene changes after Mn-ER-Cy(+) treatment, a heatmap was constructed, highlighting the differences in gene expression between the control and treated groups (Fig. 3b). To reveal the functional categories of upregulated DEGs, Kyoto Encyclopedia of Genes and Genomes (KEGG) and Gene Ontology (GO) enrichment analyses were conducted. GO analysis indicated that DEGs induced by the Mn-ER-Cy(+) group are associated with various aspects of biological processes, cellular components, and molecular functions (Fig. 3c). GO analysis showed the top five most affected biological processes: response to ERS, response to topologically incorrect protein, unfolded protein response (UPR), cellular response to topologically incorrect protein, and cellular response to unfolded protein (Fig. 3c). In addition, KEGG analysis showed that the signaling pathway of protein processing in the ER was significantly affected in the Mn-ER-Cy(+) group (Fig. 3d). Gene Set Enrichment Analysis results showed that ERS and the UPR were significantly enriched (Fig. S16). This study also analyzed which ERS-related genes were affected by Mn-ER-Cy(+) treatment. As shown in Figs. 3e and 31 genes were affected, including Ddit3 (C/EBP homologous protein [CHOP]), Hspa5 (glucose-regulated protein 78 [GRP78]), Eif2ak3 (also known as PERK), Atf3, and Atf4. These results indicated that Mn-ER-Cy(+) induces UPR and ERS.

Based on the results of transcriptome sequencing, this study further investigated in vitro whether Mn-ER-Cy-mediated CDT/PTT/PDT can disrupt ER homeostasis and induce excessive ERS. First, morphological changes in the ER in each treatment group were observed using TEM. Results showed that the ER in the control and laser groups was relatively short and appeared generally normal, whereas the ER was significantly expanded and highly convoluted in the Mn-ER-Cy group, indicating that ERS enhanced the protein folding capacity by increasing the ER surface area (Fig. 3f). Second, in the Mn-ER-Cy(+) group, the ER was almost completely depleted, indicating that excessive ERS had occurred, resulting in a significant disruption of the ER structure (Fig. 3f). Third, CHOP and GRP78 are widely recognized biomarkers for ERS [27]. Specifically, GRP78 acts as a molecular chaperone in the ER, significantly increasing its expression during ERS to assist in the folding of misfolded proteins [48]. In contrast, CHOP, a transcription factor in the UPR signaling pathway, is upregulated during prolonged and severe ERS, playing a key role in regulating cellular responses and modulating cell fate [49]. As shown in Fig. 3g -i, compared with the control group, CHOP and GRP78 expression in the Mn-ER-Cy and Mn-ER-Cy(+) groups significantly increased, with a more pronounced upward trend observed in the Mn-ER-Cy(+) group, further confirming that this treatment regimen can induce ERS in tumor cells. Fourth, the ER, a crucial reservoir for Ca^2+^ within the cell, releases a significant amount of Ca^2+^ into the cytoplasm during ERS [50]. The results indicated that the control group exhibited only weak green fluorescence signals, aligning with the fact that Ca^2+^ concentrations are relatively low under physiological conditions. In contrast, the Mn-ER-Cy and Mn-ER-Cy(+) groups showed significantly enhanced green fluorescence signals, with the Mn-ER-Cy(+) group displaying the strongest fluorescence (Fig. S17). This suggested a substantial accumulation of Ca^2+^ in the cytoplasm, which coincided with the increased Ca^2+^ release observed during ERS. Based on previous studies, each modality of CDT, PDT, and PTT can exert antitumor effects by inducing ERS [51, 52, 53]. Therefore, the Mn-ER-Cy-mediated CDT/PDT/PTT multimodal treatment would synergistically damage the ER structure and function, inducing excessive ERS.

**Fig. 4:**
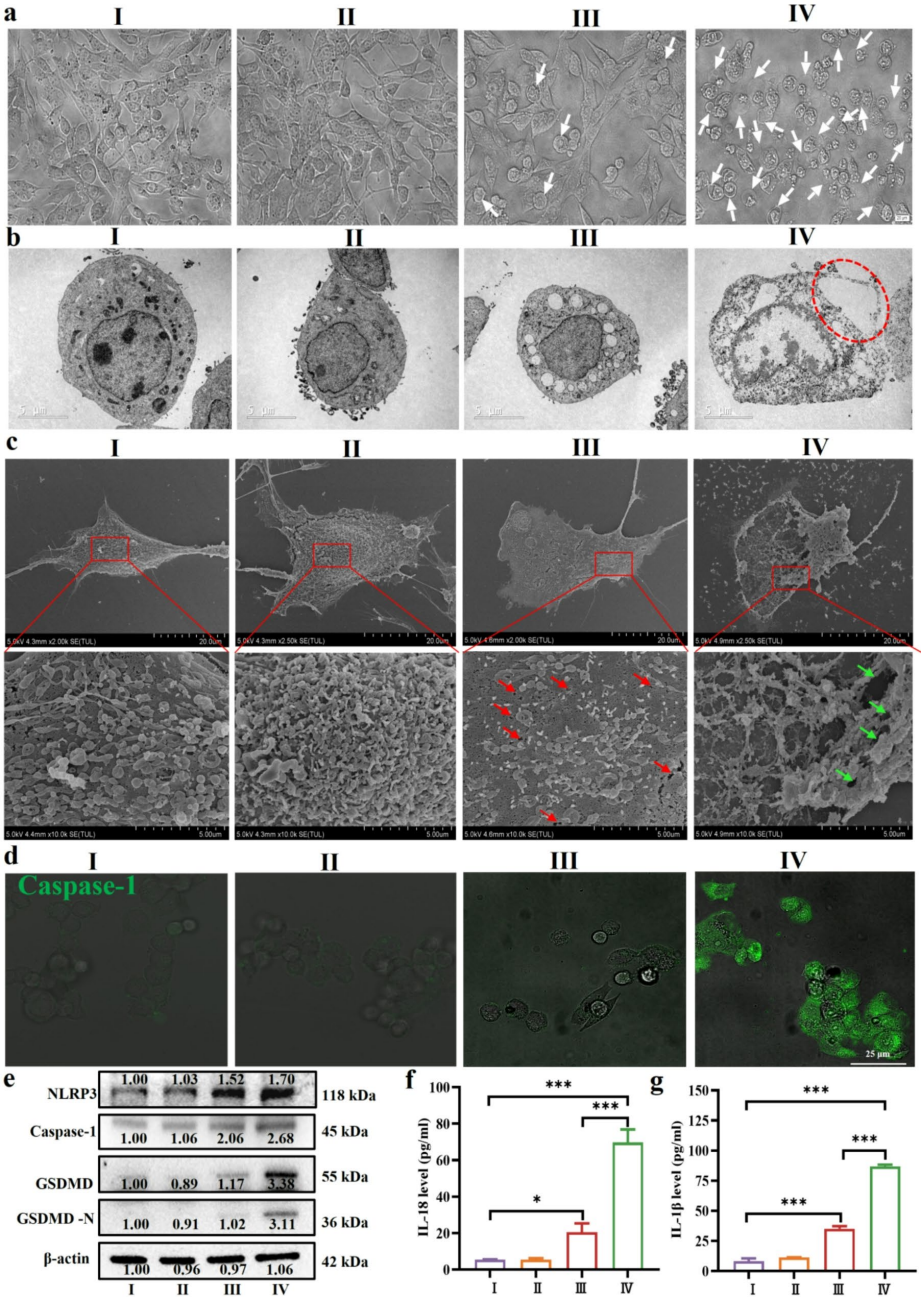
Mn-ER-Cy plus laser irradiation (+) lead to pyroptotic cell death. (**a**) Bright-field images showing 4T-1 cells morphology in each group. White arrows indicate cell swelling characterized by large bubbles. (**b**) and (**c**) Bio-TEM and SEM images illustrating the morphological features of pyroptosis. Red circles highlight large bubbles within the cells and indicate cell membrane rupture. Green arrows show cell membrane degradation and an increase in surface gaps. (**d**) CLSM images illustrating caspase-1 expression in 4T-1 cells. (**e**) Western blotting analysis of the relative protein levels of pyroptosis-related genes (NLRP3, caspase-1, GSDMD, and GSDMD-N). (**f**) and (**g**) Quantitative analysis of IL-1β and IL-18 in the cell supernatants of each group using ELISA (*n* = 3, mean ± s.d.). The experimental groups were categorized as follows: (I) PBS, (II) 808 nm laser (0.8 W/cm^2^, 5 min), (III) Mn-ER-Cy, and (IV) Mn-ER-Cy with 808 nm laser. Statistical analyses were conducted using one-way ANOVA followed by Tukey’s test. *p < 0.05, ***p < 0.001

Recent studies have indicated that ERS can induce pyroptosis by activating multiple signaling pathways, including IRE1, PERK, and ATF6 [54]. Specifically, ERS enhances CHOP expression via the PERK and eIF2α pathways, which in turn promotes NLRP3 production and further induces pyroptosis. IRE1α activates the thioredoxin-interacting protein–NLRP3 pathway, leading to the release of GSDMD protein and worsening of pyroptosis [30, 55]. Furthermore, 4-phenylbutyric acid, an ERS inhibitor, and MKC-394, an IRE1α inhibitor, have been shown to alleviate ERS and decrease pyroptosis [56]. Therefore, this study further explored whether Mn-ER-Cy(+)-induced cell death is related to the pyroptosis pathway. Similarly, the results showed that after 24 h of Mn-ER-Cy(+) treatment, most cells exhibited typical characteristics of pyroptosis, including bubble or vacuole formation, cell swelling, cell and nuclear membrane rupture, and cellular content release (Fig. 4a-c). During pyroptosis, activated NLRP3 is a sensor receptor that assembles an inflammasome, recruiting and activating caspase-1. Caspase-1 cleaves GSDMD, leading to pore formation in the cell membrane, which increases membrane permeability and facilitates cellular content release [57]. Immunofluorescence assays for caspase-1 demonstrated minimal green fluorescence signals in the control group, whereas a pronounced green fluorescence signal was observed in the Mn-ER-Cy(+) group (Fig. 4d), indicating an elevated caspase-1 expression in the treated cells. Moreover, western blotting analysis further confirmed that Mn-ER-Cy(+) treatment significantly elevated the levels of NLRP3, caspase-1, and GSDMD-N (Fig. 4e). Caspase-1 also promotes the maturation and release of proinflammatory cytokines such as interleukin (IL)-1β and IL-18, initiating a robust inflammatory response [15]. Consistently, enzyme-linked immunosorbent assay indicated that compared with the control group, IL-1β and IL-18 levels in the cell supernatant of the Mn-ER-Cy(+) group were significantly elevated, with increases of 10-fold (from 8.28 to 86.95 pg/mL) and 12-fold (from 5.45 to 69.81 pg/mL), respectively (Fig. 4f and g). In addition, both the NLRP3 inhibitor (MCC950) and NLRP3 siRNA effectively attenuated Mn-ER-Cy (+)-induced pyroptotic effects, as evidenced by the alleviation of typical pyroptotic cellular morphology, reduced membrane rupture, and decreased expression of pyroptosis-related proteins (Fig. S18-19). These results demonstrated that Mn-ER-Cy(+) NCs effectively induced cell pyroptosis in vitro, and the underlying mechanism may be associated with the activation of the NLRP3/caspase-1/GSDMD signaling pathway mediated by ERS.

### Mn-ER-Cy neutralized the acidic TME and augmented ICD

**Fig. 5:**
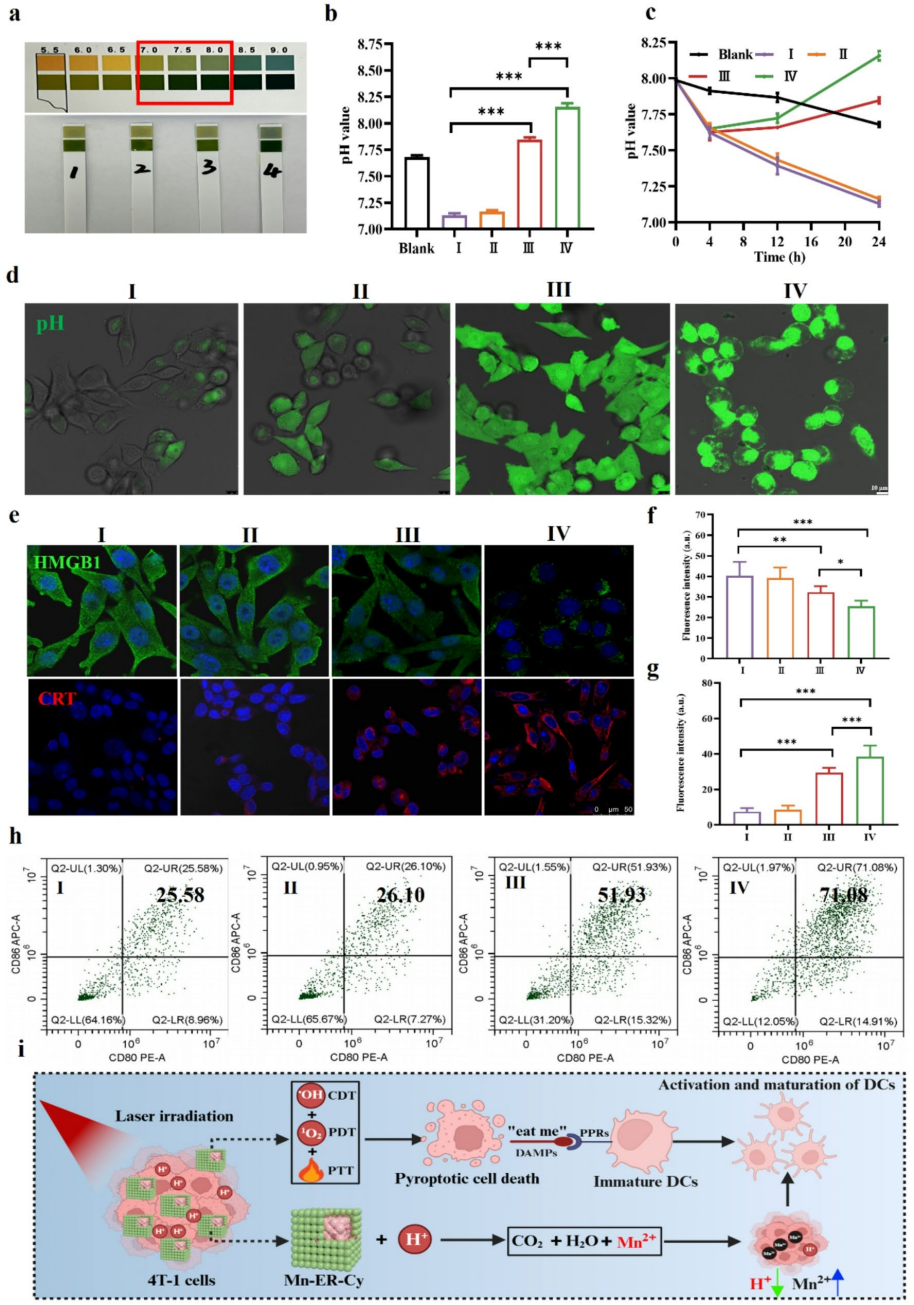
Mn-ER-Cy neutralized the acidic TME and enhanced ICD. (**a**) and (**b**) pH test paper and a pH meter revealing the pH values of cell supernatants after various treatments for 24 h (*n* = 3, mean ± s.d.). (**c**) Time-dependent pH change curves for cell supernatants in each group (*n* = 3, mean ± s.d.). (**d**) CLSM images showing the intracellular pH values of cells after various treatments for 24 h. Green fluorescence intensity correlates with higher pH. (**e**) CLSM images showing CRT and HMGB1 expression in cells after various treatments for 24 h. (**f**) and (**g**) semiquantitative analysis of the fluorescence intensities of CRT and HMGB1 from e (*n* = 3, mean ± s.d.). (**h**) Flow cytometry analysis of BMDC maturation after incubation with supernatants from each treatment group for 24 h. (**i**) Schematic diagram illustrating the potential mechanism by which Mn-ER-Cy promotes DC activation and maturation. The experimental groups were categorized as follows: (I) PBS, (II) 808 nm laser (0.8 W/cm^2^, 5 min), (III) Mn-ER-Cy, and (IV) Mn-ER-Cy with 808 nm laser. Statistical analyses were conducted using one-way ANOVA followed by Tukey’s test. *p < 0.05, **p < 0.01, ***p < 0.001

Accumulating evidence indicated that the acidic TME promotes tumor proliferation, apoptosis evasion, invasion, metastasis, and angiogenesis through various mechanisms [9]. Acidic TME is a major contributor to tumor immune evasion because it significantly suppresses the function of immune cells, including T cells, DCs, and macrophages [58]. For instance, low pH can reduce T cell proliferation and cytotoxicity, potentially resulting in immune tolerance [59]. It also impairs DCs, diminishing their capacity to present antigens. Furthermore, macrophages are more likely to shift toward the M2 phenotype in an acidic environment, which inhibits antitumor immune responses. Therefore, neutralizing the acidic TME is an important strategy for inhibiting tumor growth and stimulating antitumor immunity. Considering that MnCO_3_ easily decomposes into Mn^2+^, H_2_O, and CO_2_ in acidic environments, consuming H^+^, this study further evaluated whether Mn-ER-Cy can reverse the acidic TME. As shown in Fig. 5a, after 24 h of treatment, Mn-ER-Cy significantly increased the pH of the cell supernatant compared with the control group, which may primarily be attributed to the acid-consuming effect of MnCO_3_. In particular, Mn-ER-Cy combined with laser irradiation showed a significant increase in pH compared with Mn-ER-Cy alone (Fig. 5b), suggesting a potential for effectively neutralizing acid TME by light irradiation in a more specific and controllable manner.

This study further explored the relationship between the effect of Mn-ER-Cy on pH and incubation time. Results showed that in the first 4 h, the pH of cell supernatants in all groups decreased, which may be related to the accumulation of acidic metabolic products (such as lactate) produced by tumor cells during metabolism and the dissolution of CO_2_ in the culture medium. In contrast, after 4 h, Mn-ER-Cy group began to increase and the Mn-ER-Cy combined with the laser group showed a more pronounced trend (Fig. 5c and Fig. S20). This phenomenon again demonstrated that Mn-ER-Cy was substantially taken by tumor cells and could neutralize the acid environment, particularly when combined with laser irradiation. Furthermore, the intracellular pH (pHi) change was examined using a pH-sensitive fluorescent probe, BBcellProbe^®^ P01. As expected, the control and laser groups exhibited only weak green fluorescence, indicating a low pHi associated with an acidic environment (Fig. 5d). In contrast, the Mn-ER-Cy and Mn-ER-Cy combined with the laser groups showed strong green fluorescence signals, suggesting a high pHi indicative of a slightly alkaline environment.

Unlike other cell death mechanisms, such as apoptosis and necrosis, pyroptosis is a highly immunogenic form of cell death [14]. During this process, calreticulin (CRT) translocates from the ER to the cell surface, serving as an “eat me” signal to recruit immune cells. Meanwhile, high mobility group protein 1 (HMGB1) is released from the nucleus into the extracellular space, acting as a danger signal that promotes immune response activation and enhancement [60]. Therefore, the effects of Mn-ER-Cy on the expression and distribution of CRT and HMGB1 in cells was assessed using immunofluorescence. As shown in Fig. 5e-g, compared with the control group, the Mn-ER-Cy and Mn-ER-Cy(+) groups showed increased CRT expression, whereas intracellular HMGB1 decreased significantly, with changes being more pronounced in the Mn-ER-Cy(+) group. Previous studies showed that Mn^2+^-mediated Fenton-like reactions can generate ·OH by reacting with H_2_O_2_ within tumor cells, killing the cells and inducing ICD [61]. Therefore, CRT expression and HMGB1 release induced by Mn-ER-Cy might be mediated by the CDT effects of Mn^2+^. CDT/PDT/PTT multimodal approaches can exhibit a synergistic effect in inducing ICD.

During pyroptosis, cells release various DAMPs and antigens that activate DCs, promoting antigen presentation and enhancing T cell activation and proliferation to effectively recognize and attack tumor cells [62]. To validate this process, the impact of conditioned medium from each treatment group on the maturation of bone marrow-derived DCs (BMDCs) was assessed. Consistent with the abovementioned findings, flow cytometry analysis revealed that the proportion of mature BMDCs (CD11c^+^-CD80^+^-CD86^+^) in the control group was 25.58%. However, the proportions in the Mn-ER-Cy group (51.93%) and Mn-ER-Cy(+) group (71.08%) were significantly higher, with the combination group exhibiting even more pronounced effects (Fig. 5h and i). These results suggest that Mn-ER-Cy mediates multimodal cancer therapies, including CDT, PDT, and PTT, which can effectively induce ICD in tumor cells and promote DCs maturation.

### Tumor Preferential accumulation and dual-modality imaging

**Fig. 6:**
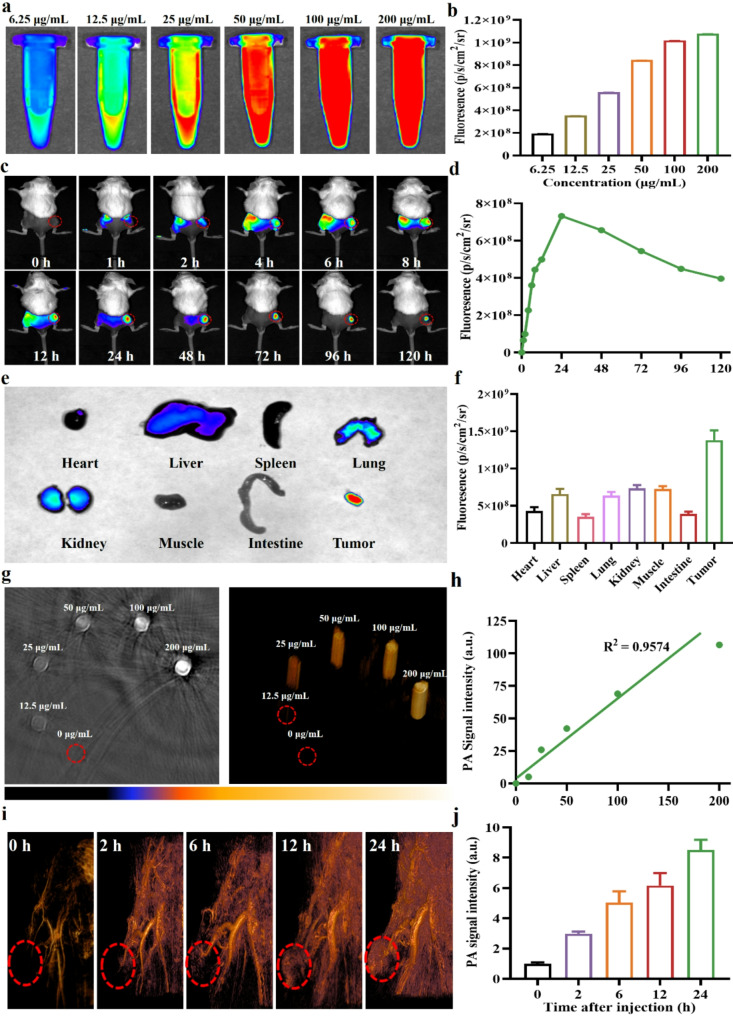
Tumor-targeting accumulation and NIR/PAI dual-modality imaging. (**a**) NIR fluorescence imaging of Mn-ER-Cy at different concentrations. (**b**) Semiquantitative analysis of fluorescence intensities from (a). (**c**) NIR fluorescence imaging analysis of Mn-ER-Cy accumulation in tumor regions at various time intervals. Red circles represent tumor regions. (**d**) Semiquantitative analysis of fluorescence intensities in tumor tissues from (c). (**e**) Ex vivo NIR fluorescence imaging of different organs and tissues (liver, heart, spleen, lungs, kidneys, muscle, and tumor) collected from mice 24 h after Mn-ER-Cy injection. (**f**) Semiquantitative analysis of fluorescence intensities in vital organs and tissues (*n* = 3, mean ± s.d.). (**g**) In vitro PAI of Mn-ER-Cy at varying concentrations under 808 nm laser irradiation. Left, axial slice; right, 3D view. (**h**) Linear relationship between Mn-ER-Cy concentrations and PAI signal intensities. (**i**) PAI analysis of Mn-ER-Cy accumulation in tumor regions at various intervals. Red circles represent tumor regions. (**j**) Semiquantitative analysis of PAI signal intensities in tumor tissues from (i), with PAI signal intensities at 0 h serving as the baseline (*n* = 3, mean ± s.d.)

Tumor-targeted accumulation and multimodal imaging are essential for achieving precise tumor diagnosis and enhancing therapeutic efficacy [63]. Accordingly, the tumor-targeting and imaging capabilities of Mn-ER-Cy were evaluated in a 4T-1 tumor-bearing mouse model. Mn-ER-Cy exhibited strong NIR fluorescence (NIRF) emission, which increased with enhanced drug concentrations (Fig. 6a-b). Mn-ER-Cy was injected into 4T-1 tumor-bearing mice, and tumor-targeting accumulation was recorded using a NIR small-animal imaging system. The results indicated that after Mn-ER-Cy injection, fluorescence signals at the tumor site began to appear after 2 h, gradually increasing over time and peaking at 24 h (Fig. 6c and d). Meanwhile, as signals in other areas gradually diminished, the tumor site maintained a high fluorescence signal even at 120 h after injection, suggesting that Mn-ER-Cy can rapidly and persistently accumulate at the tumor site to facilitate sustained antitumor effects. Consistent with in vivo results, ex vivo results at 24 h showed that the fluorescence signal at the tumor site was significantly stronger than that in other organs and tissues (heart, liver, spleen, lung, kidney, muscle, and intestine) (Fig. 6e and f)).

Subsequently, the photoacoustic imaging (PAI) capabilities of Mn-ER-Cy were also evaluated in vitro and in vivo. As depicted in Fig. 6g, Mn-ER-Cy can generate a significant PAI signal even at a low concentration of 12.5 µg/mL. Furthermore, signal intensity increases gradually with higher concentrations, demonstrating a strong linear relationship (Fig. 6h). This suggested that Mn-ER-Cy is effective for PAI across a range of concentrations, indicating excellent imaging performance and sensitivity. In addition, in vivo PAI showed that Mn-ER-Cy can effectively accumulate in the tumor site, reaching peak signal intensity after 24 h, clearly indicating the tumor region (Fig. 6i-j). Therefore, these findings suggested that Mn-ER-Cy possesses excellent tumor-targeting accumulation properties and NIR/PAI dual-modality imaging capabilities, demonstrating their potential as a highly sensitive contrast agent to support imaging-guided tumor diagnosis and therapy.

### Mn-ER-Cy suppressed primary tumor growth by eliciting antitumor immunity

Inspired by the positive antitumor effect of Mn-ER-Cy observed in vitro, this study further investigated their antitumor activity in vivo. The 4T-1 tumor-bearing mice were randomly assigned to four groups to assess their effects on tumor growth: (I) control group, (II) laser group, (III) Mn-ER-Cy group, and (IV) Mn-ER-Cy(+) plus laser group. The tumor-site temperature of mice in the control group only increased 5 °C after 5 min laser irradiation. In contrast, the tumor-site temperature in the Mn-ER-Cy(+) group showed a dramatic increase (ΔT = 23 °C) (Fig. S21). This further indicated the effective intratumoral accumulation of Mn-ER-Cy and its excellent PCE in tumors. The results indicated that the control and laser groups exhibited the fastest tumor growth and largest tumor volumes (Fig. S22a-c). The Mn-ER-Cy group showed moderate tumor inhibition, likely attributed to the beneficial effect of mild CDT alone. In contrast, the Mn-ER-Cy(+) group exhibited the smallest tumor volume, owing to the combined therapeutic effects of CDT, PDT, and PTT. Most importantly, no significant weight loss was observed in mice in any group during the treatment period, suggesting that the multi-model treatments are tolerable for mice (Fig. S22d). Hematoxylin-and-eosin (H&E) staining of tumor tissues showed significant cell damage and necrosis in the Mn-ER-Cy(+) group (Fig. S23a). Among the groups, the proportion of Ki-67^+^ cells was the lowest in the Mn-ER-Cy(+) group, whereas the number of terminal deoxynucleotidyl transferase-mediated dUTP nick-end labeling-positive cells was the highest (Fig. S23a-c). These data suggest that Mn-ER-Cy-mediated CDT/PDT/PTT can effectively suppress tumor growth with minimal adverse effects.

**Fig. 7:**
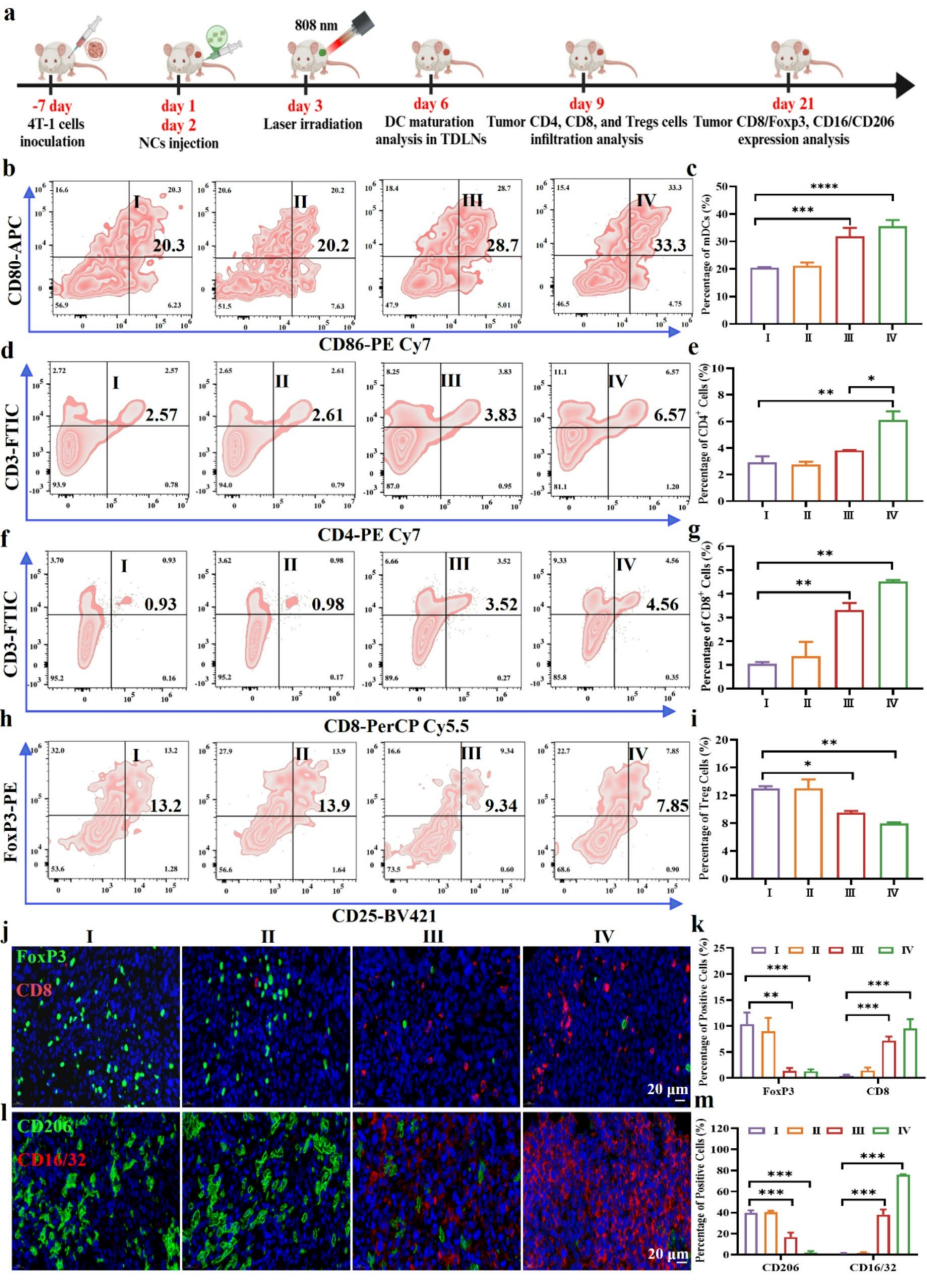
In vivo immunostimulation effects. (**a**) Schematic diagram illustrating the procedures for the in vivo immunoassay. (**b**) Flow cytometry analysis of DC maturation (CD80⁺–CD86⁺) in tumor-draining lymph nodes 3 days after the final treatment. (**c**) Quantitative analysis of the proportion of CD80^+^-CD86^+^ cells from (b) (*n* = 3, mean ± s.d.). (**d**, **f**, **h**) Flow cytometry analysis of the infiltration of CD4^+^ T cells (CD3^+^–CD4^+^), CD8^+^ T cells (CD3^+^–CD8^+^), and Treg cells (CD25^+^–Foxp3^+^) in tumor tissues 6 days after the final treatment. (**e**, **g**, **i**) Quantitative analysis of the proportion of CD3^+^-CD4^+^, CD3^+^-CD8^+^, and CD25^+^-Foxp3^+^ cells in tumor tissues (*n* = 3, mean ± s.d.). (**j**) and (**l**) Immunofluorescence analysis of CD8, FOXP3, CD16/CD32, and CD206 expression in tumor tissues 21 days after treatment. (**k**-**m**) Quantitative analysis of the proportion of CD8^+^, Foxp3^+^, CD16/CD32^+^, and CD206^+^ cells in tumor tissues. The experimental groups were categorized as follows: (I) PBS, (II) 808 nm laser (0.5 W/cm^2^, 5 min), (III) Mn-ER-Cy, and (IV) Mn-ER-Cy with 808 nm laser. Statistical analyses were conducted using one-way ANOVA followed by Tukey’s test. *p < 0.05, **p < 0.01, ***p < 0.001

Pyroptotic cell death can release inflammatory factors and DAMPs, enhancing the local immune response and promoting DC activation and maturation [64]. Furthermore, neutralizing the acidic TME can improve the functional status of DCs, enhancing their ability to present antigens and stimulate T cells [65]. Therefore, this study first evaluated the effects of Mn-ER-Cy on DC maturation in tumor-draining lymph nodes (Fig. 7a). Consistent with the existing literature, results indicated that the DC maturation rate in the control group (CD86^+^-CD80^+^) was 20.3%, whereas the rates in the Mn-ER-Cy and Mn-ER-Cy(+) groups significantly increased to 28.7% and 33.3%, respectively (Fig. 7b-c). Mature DCs play a crucial role in the activation and proliferation of T cells. Mature DCs provide necessary costimulatory signals by interacting with T cell surface receptors, such as CD28, through surface costimulatory molecules, such as CD80 and CD86, promoting the activation and proliferation of T cells. Conversely, they also effectively present antigens by displaying antigen fragments to T cells via major histocompatibility complex molecules, thus stimulating a specific immune response. As expected, the proportions of CD3^+^-CD4 + and CD3^+^-CD8^+^ T cells in the Mn-ER-Cy(+) group increased to 6.57% and 4.56%, respectively, which were 2.5 times (2.57%) and 5 times (0.93%) that of the control group (Fig. 7d-g). These data suggested that Mn-ER-Cy may recruit immune cells and activate adaptive immune responses.

Immunosuppressive cells in the TME, such as Tregs, significantly reduce the activity of effector T cells and other immune cells by releasing inhibitory cytokines, leading to the poor efficacy of immunotherapy. Results indicated that Mn-ER-Cy(+) treatment could also reduce the proportion of Tregs in the TME, with the proportion of CD25^+^-Foxp3^+^ cells being 13.2% in the control group and 7.85% in the Mn-ER-Cy(+) group (Fig. 7h-i). The acidic TME significantly affects Treg proliferation and activity through various mechanisms, including surface marker expression, metabolic reprogramming, signaling pathways, and cell interactions. For example, acidic TME can promote the upregulation of inhibitory molecules, such as CTLA-4 and PD-1, thus enhancing Treg immunosuppression and inhibiting effector T cell activity. Therefore, Treg reduction mediated by Mn-ER-Cy(+) may be partially attributed to its ability to correct the acidic TME. In addition, results of dual CD8-Foxp3 staining in tumor tissues showed a similar trend, with many Foxp3^+^ cells (green fluorescence) and fewer CD8 + cells in the control group. In contrast, in the Mn-ER-Cy(+) group, green fluorescence significantly decreased, whereas red fluorescence increased (Fig. 7j-k).

TAMs are critical constituents of the TME, exhibiting multifaceted roles in tumorigenesis and progression. Specifically, M1 macrophages are typically stimulated by proinflammatory factors and exhibit strong antitumor activity, secreting proinflammatory cytokines such as tumor necrosis factor-α and IL-12, which enhance antigen presentation and T cell activation [66]. In contrast, M2 macrophages, which are induced by anti-inflammatory factors and specific stimuli in the TME, secrete cytokines, such as IL-10 and transforming growth factor-β, which significantly promote tumor growth, metastasis, and immune suppression [67]. These findings revealed that Mn-ER-Cy treatment can influence the polarization pattern of TAMs, promoting the conversion of immunosuppressive M2 phenotype macrophages to the cytotoxic M1 phenotype (Fig. 7l-m). Similarly, numerous studies showed that CDT/PDT/PTT multimodal therapy and correcting the acidic TME can effectively reprogram macrophages from the M2 to the M1 phenotype [10]. In addition to mediating the CDT effect, several studies demonstrated that Mn^2+^ enhances DC maturation and macrophage repolarization by activating the cGAS-STING pathway [68]. This process significantly promotes the infiltration of cytotoxic T lymphocytes and helper T cells, which remodels the TME and inhibits tumor growth and metastasis [69]. Previous studies supported the idea that Mn-ER-Cy treatment can effectively enhance tumor adaptive immunity and improve the immunosuppressive acidic TME, providing a favorable environment for amplifying systemic antitumor immunity.

### Mn-ER-Cy suppressed the growth of distant tumors and lung metastasis

**Fig. 8:**
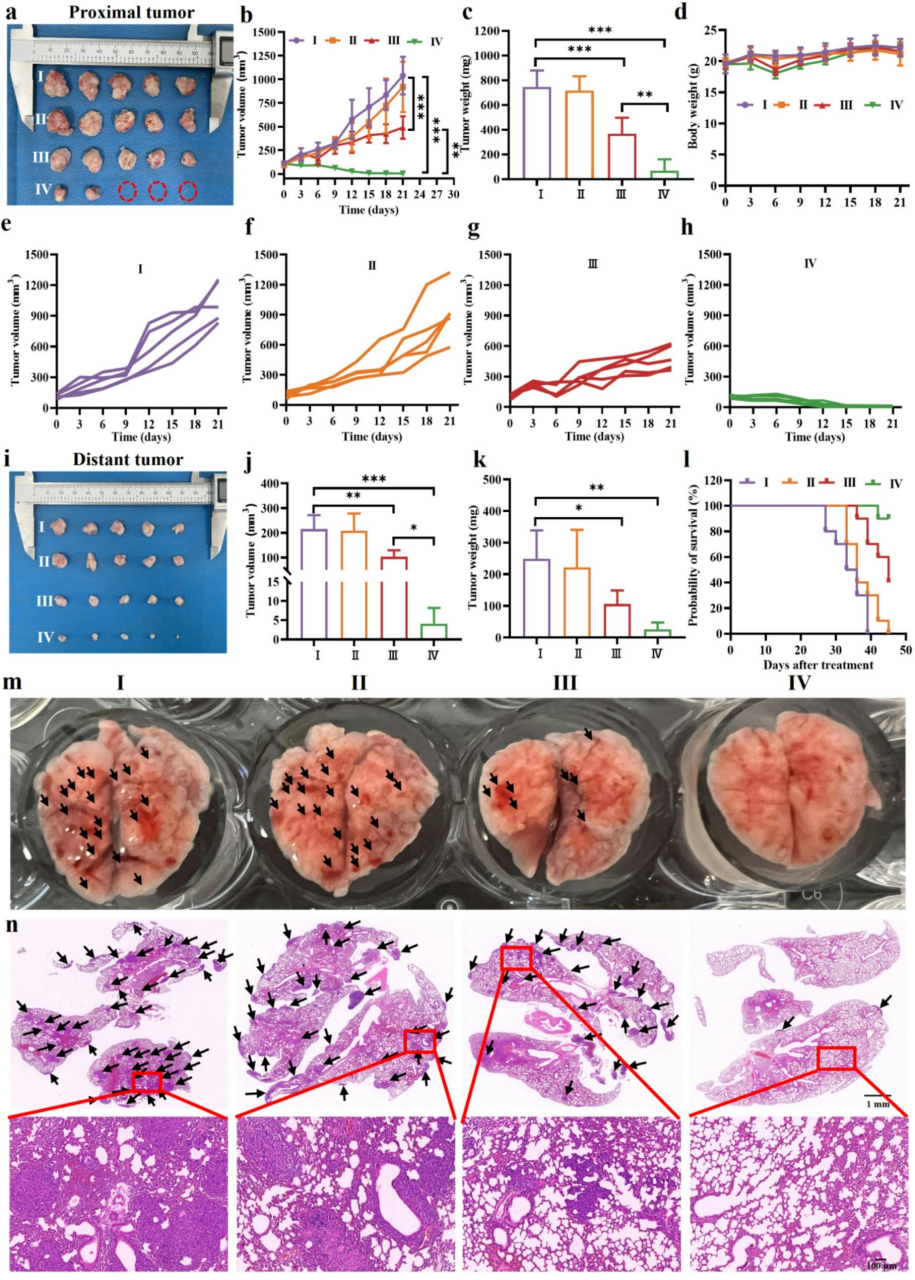
Mn-ER-Cy inhibited tumor growth and metastasis in vivo. (**a**) Images of proximal tumors and (**b**) tumor volume curves on day 21 after various treatments (*n* = 5, mean ± s.d.). (**c**) Tumor weights and (**d**) body weights during the treatment period (*n* = 5, mean ± s.d.). (**e**-**h**) Tumor growth curves for individual tumors in mice across various groups. Images of (**i**) distant tumors and (**j** and **k**) tumor volume and weights on day 21 after various treatments (*n* = 5, mean ± s.d.). (**l**) Survival analysis of mice in each group during the 45-day observation period after treatment (*n* = 10). (**m**) Representative pictures of mouse lung metastasis on day 21. (**n**) Representative H&E images depicting lung metastasis nodules in each group. Black arrows indicate lung metastasis nodules. The experimental groups were categorized as follows: (I) PBS, (II) 808 nm laser (0.5 W/cm^2^, 5 min), (III) Mn-ER-Cy, and (IV) Mn-ER-Cy with 808 nm laser. Statistical analyses were conducted using one-way ANOVA followed by Tukey’s test. *p < 0.05, **p < 0.01, ***p < 0.001

Inspired by its promising immunostimulatory effects, this study next investigated whether Mn-ER-Cy can inhibit the growth of distal tumors by stimulating the body’s immune response in a bilateral tumor model. The results demonstrated that Mn-ER-Cy(+) effectively inhibited primary tumor growth (Fig. 8a-h), with the tumor growth inhibition index reaching as high as 80%. More impressively, the Mn-ER-Cy(+) group demonstrated the smallest volume of distal tumors (< 5 mm^3^), whereas the control group exhibited tumor volumes exceeding 200 mm^3^, resulting in a tumor growth inhibition index of 70% (Fig. 8i-k). In addition, Mn-ER-Cy(+) treatment afforded a 90% survival rate in tumor-bearing mice (Fig. 8l).

The lungs are one of the most frequent sites of metastasis in breast cancers. In patients with advanced breast cancer, lung metastasis incidence is estimated to range from 30 to 50%, with certain subtypes, such as HER-2 + and triple-negative breast cancer, exhibiting an even higher prevalence [70]. The anti-metastatic effect of Mn-ER-Cy were evaluated in a lung metastasis model. The appearance of lung tissue images indicated that the control and laser groups exhibited many metastatic nodules. In the Mn-ER-Cy group, the number of nodules was relatively reduced, which may be attributed to CDT effects (Fig. 8m). Furthermore, the Mn-ER-Cy(+) group showed the least number of lung metastatic nodules, and the morphology of the lung tissue appeared relatively normal, indicating a synergistic inhibitory effect of CDT/PDT/PTT on tumor metastasis facilitated by Mn-ER-Cy. Moreover, H&E staining results of the lung tissue further supported this observation, revealing a similar trend (Fig. 8n). These findings indicated that Mn-ER-Cy not only inhibits primary tumor growth but also enhances systemic antitumor effects by activating the body’s adaptive immune response, thus effectively suppressing tumor metastasis.

### Pharmacokinetics and safety evaluation

Biological safety is a key factor limiting the clinical translation of nanomedicine [71]. Therefore, this study evaluated the pharmacokinetics and biocompatibility of Mn-ER-Cy in healthy mice. Ex vivo fluorescence imaging of the organs showed that Mn-ER-Cy primarily accumulated in the liver (Fig. S24a). The fluorescence intensity in the liver peaked within 8 h and gradually decreased, indicating that Mn-ER-Cy can be rapidly absorbed and metabolized in the liver (Fig. S24c). Similarly, blood fluorescence intensity indicated that after intraperitoneal administration, fluorescence signals appeared at 1 h, peaked within 8 h, and gradually weakened (Fig. S24b and d), demonstrating the rapid absorption and distribution of Mn-ER-Cy in the body. This study also assessed the blood compatibility of Mn-ER-Cy. Negligible hemolysis was observed at low-to-medium concentrations (< 100 µg/mL) (Fig. S25). Although some hemolysis (3.3%) was noted at a high concentration (400 µg/mL), it was still significantly lower than the National Medical Products Administration standard threshold of 5.0% set for the drug hemolysis rate, indicating excellent blood compatibility. Next, the effects of different doses of Mn-ER-Cy treatment (0, 10, 20, and 40 mg/kg) on the blood routine, liver and kidney function, and organ damage in normal mice were evaluated after 21 days. Compared with the control group, there were no significant differences in the parameters of blood routine (white blood cells, red blood cells, and platelets) and liver, kidney and heart function (alanine transaminase, aspartate transaminase, urea, creatinine, lactate dehydrogenase, and creatine kinase) among the treatment groups, all of which remained within the normal threshold range (Fig. S26 and Fig. S27). Furthermore, H&E staining analysis of tissues and organs (heart, liver, spleen, lung, and kidney) from each group of mice showed no significant structural damage or inflammatory cell infiltration (Fig. S28). These data suggest that Mn-ER-Cy possesses excellent specificity and biocompatibility, providing a potential for further translational research.

As summarized in Table S2, most previously reported Mn-based nanoplatforms primarily rely on passive tumor accumulation via the enhanced permeability and retention (EPR) effect, exhibiting limited subcellular targeting capabilities. In contrast, Mn-ER-Cy enables active and precise targeting of tumor-cell ER organelles, thereby facilitating localized therapeutic effects. Notably, Mn-ER-Cy achieves a significantly higher photothermal conversion efficiency (51.6%) under 808 nm irradiation compared to conventional systems (typically below 40%). Furthermore, whereas traditional Mn-based nanocarriers are generally confined to a single imaging modality, Mn-ER-Cy offers dual-modal imaging through NIRF and photoacoustic imaging, enabling real-time monitoring and precision-guided therapy. Therapeutically, Mn-ER-Cy integrates PTT, PDT, and CDT, collectively inducing excessive ER stress, triggering pyroptotic cell death, and augmenting antitumor immunity. These distinct advantages underscore the clinical potential of Mn-ER-Cy as a versatile platform for precision tumor theranostics.

Although Mn-ER-Cy demonstrates multifunctional properties and favorable biological performance in preliminary studies, its clinical translatability remains to be thoroughly investigated. Large-scale synthesis is theoretically feasible through the optimization of reaction conditions and purification processes; however, challenges such as batch-to-batch reproducibility, long-term stability, and cost-effectiveness must be systematically addressed. Furthermore, although initial biosafety assessments indicate low acute toxicity, comprehensive evaluations—including chronic toxicity, immunogenicity, metabolism, and excretion studies—are essential before clinical application. Future research will focus on detailed pharmacokinetics, pharmacodynamics, and evaluations in large-animal models to further substantiate the clinical potential of Mn-ER-Cy.

## Conclusions

MnCO_3_ integrated with a dual-targeting Cy photosensitizer within the Mn-ER-Cy nanocube was designed to address the current challenges of clinical immunotherapy posed by the immunosuppressive acidic TME, low immunogenicity of tumors, and poor specificity of immunoactivation. Our findings revealed that Mn-ER-Cy preferentially accumulates in tumor tissues, neutralizes acidity, and induces ERS and pyroptotic cell death through various therapeutic modalities, promoting a robust antitumor immune response and suppressing tumor growth and metastasis. Its dual-modality imaging capabilities also provide precise guidance for treatment and enable effective therapeutic monitoring. These findings underscore the potential of Mn-ER-Cy as an innovative strategy to enhance the efficacy and specificity of immunotherapy, offering promising implications for better clinical outcomes in patients with cancer.

## Electronic supplementary material

Below is the link to the electronic supplementary material.

Supplementary Material 1

Supplementary Material 2

Supplementary Material 3

Supplementary Material 4

Supplementary Material 5

## Data Availability

No datasets were generated or analysed during the current study.

## Abbreviations

BMDCs: Bone marrow derived dendritic cells
CLSM: Confocal laser scanning microscopy
CTAB: Cetyltrimethylammonium bromide
CTLs: Cytotoxic T lymphocytes
DAMPs: Danger-associated molecular patterns
DC: Dendritic cell
ER: Endoplasmic reticulum
ERS: Endoplasmic reticulum stress
GSDMD: Gasdermin D
HMGB1: High mobility group protein 1
ICD: Immunogenic cell death
ICP-MS: Inductively coupled plasma mass spectrometry
NIR: Near-infrared
PAA: Polyacrylic acid
PCE: Photothermal conversion efficiency
PTAs: Photothermal agents
TAMs: Tumor-associated macrophages
TEM: Transmission electron microscopy
TME: Tumor microenvironment
Tregs: Regulatory T cells
UPR: Unfolded protein response
