# Supplementary material for: Light-activatable manganese carbonate nanocubes elicit robust immunotherapy by amplifying endoplasmic reticulum stress-meditated pyroptotic cell death
Source: J Exp Clin Cancer Res. 2025 May 16;44:147. doi: 10.1186/s13046-025-03408-5 (PMC12082914; doi:10.1186/s13046-025-03408-5)
Supplement: Supplementary file 1 — Supplementary Material 1 [file 13046_2025_3408_MOESM1_ESM.docx]

**Supplementary Material**

**Light-Activatable Manganese Carbonate Nanocubes Elicit Robust Immunotherapy by Amplifying Endoplasmic Reticulum Stress-Meditated Pyroptotic Cell Death**

Chuan Wu^1,#^, Mingquan Gao^2,#^, Weidong Xiao^3,#^, Xie Huang^2^, Xinrui Yang^2^, Zifei Wu^2^, Xudong Yu^2^, Banghui Mo^2^, Zaizhi Du^2^, Ziqian Shang^2^, Jing Liu^2^, Can Shi^2^, Rong Li^2,^*, Shenglin Luo^2,^*, Weidong Wang^1,^*

^1^ Department of Radiation Oncology, Sichuan Clinical Research Center for Cancer, Sichuan Cancer Hospital & Institute, Sichuan Cancer Center, School of Medicine, University of Electronic Science and Technology of China, Chengdu 610054, China.

^2^ Institute of Combined Injury, State Key Laboratory of Trauma and Chemical Poisoning, Chongqing Engineering Research Center for Nanomedicine, College of Preventive Medicine, Third Military Medical University (Army Medical University), Chongqing 400038, China.

^3^ Department of Pharmacy, Daping Hospital, Third Military Medical University (Army Medical University), Chongqing 400042, China.

^#^ These authors contributed equally to this work.

*** Corresponding authors.**

Email addresses: lrong361@126.com (R. Li); luosl@tmmu.edu.cn (S. Luo); wwdwyl@uestc.edu.cn (W. Wang).

**Supplementary Figures**

| 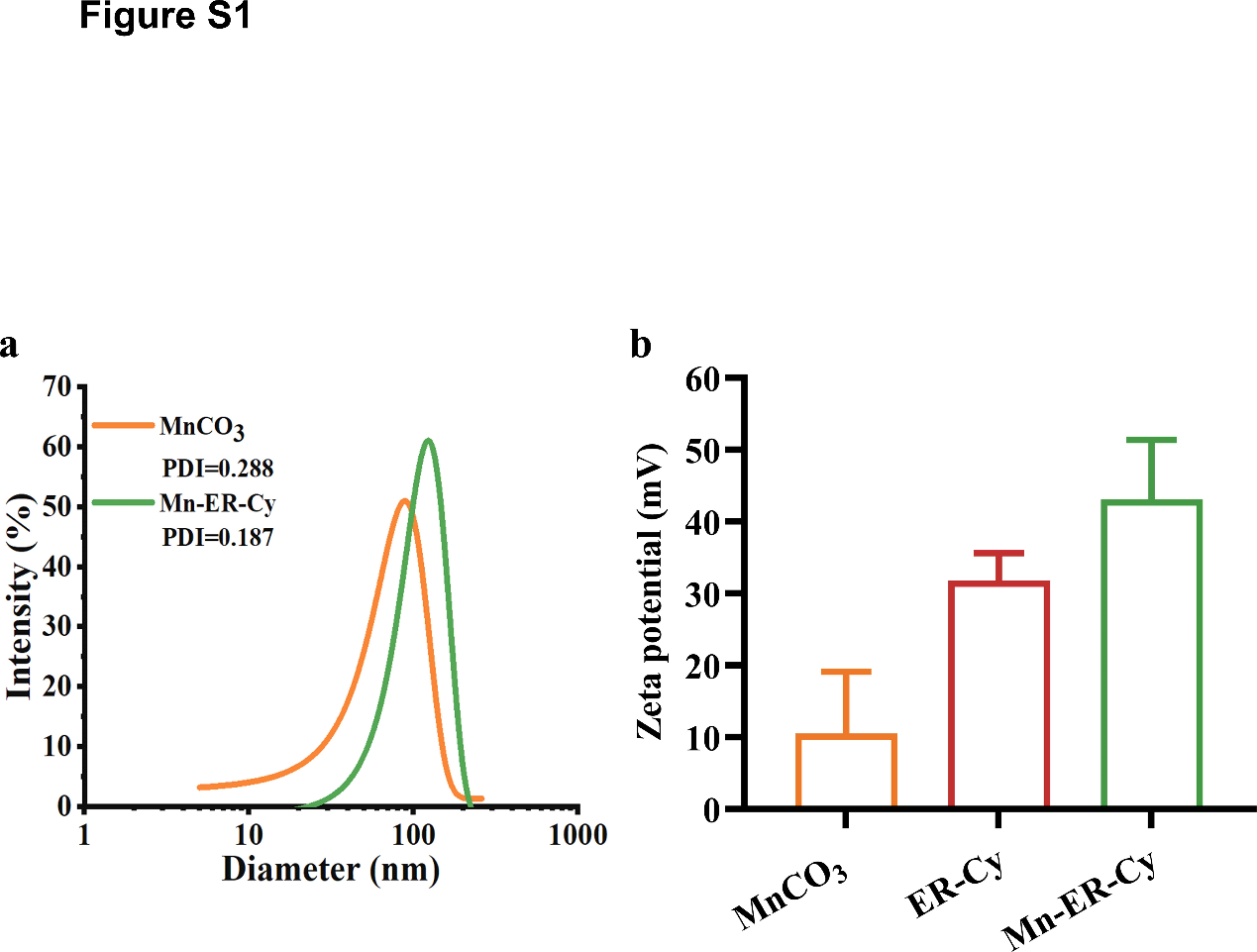 |
| --- |
| **Fig. S1** (**a**) Hydrodynamic particle size distribution of both MnCO_3_ and Mn-ER-Cy. (**b**) Zeta potential measurements for MnCO_3_, ER-Cy, and Mn-ER-Cy (n=3, mean ± s.d.). |

| 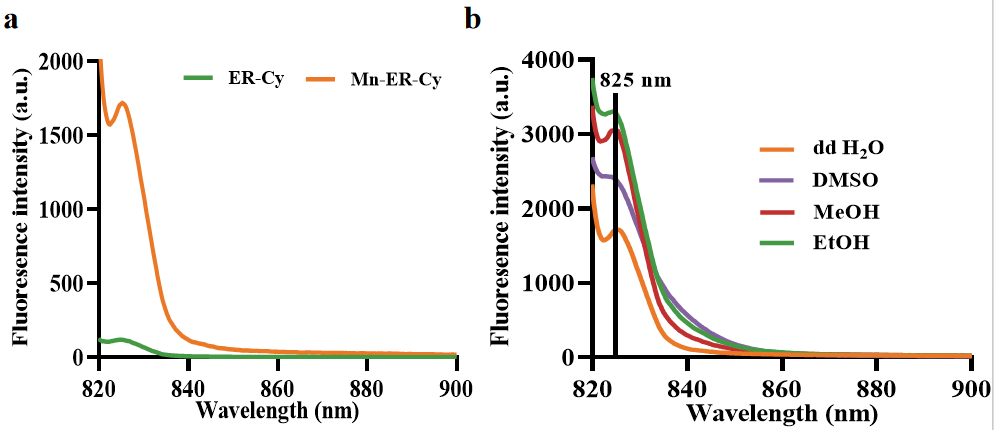 |
| --- |
| **Fig. S2** (**a**) Fluorescence emission spectrum of ER-Cy and Mn-ER-Cy in aqueous solution, with excitation at 805 nm. (**b**) Fluorescence emission spectrum of Mn-ER-Cy in different solvents, with excitation at 805 nm. A significant increase of fluorescent intensity was found for Mn-ER-Cy in aqueous solution after ER-Cy was modified on MnCO_3_-PAA nanocube, indicating a great improvement for the water-soluble ability of ER-Cy. |

|  |
| --- |
| **Fig. S3** (**a**) TEM characterization of the morphological changes of Mn-ER-Cy after prolonged storage. (**b**-**c**) DLS analysis of the variations in hydrodynamic size and polydispersity index (PDI) of Mn-ER-Cy during 21 days of storage in PBS. (**d**) UV/Vis/NIR absorption spectra of Mn-ER-Cy in PBS solution. (**e**) The cumulative release profile of Mn²⁺ from Mn-ER-Cy in PBS solution at different pH values. |

|  |
| --- |
| **Fig. S4** (**a**) CLSM images illustrating the intracellular uptake of Mn-ER-Cy in 4T-1 cells at various time points. Scale bar = 10 µm. (**b**) Semi-quantitative analysis of fluorescence intensity shown in Fig. a (n=5, mean ± s.d.). |

| 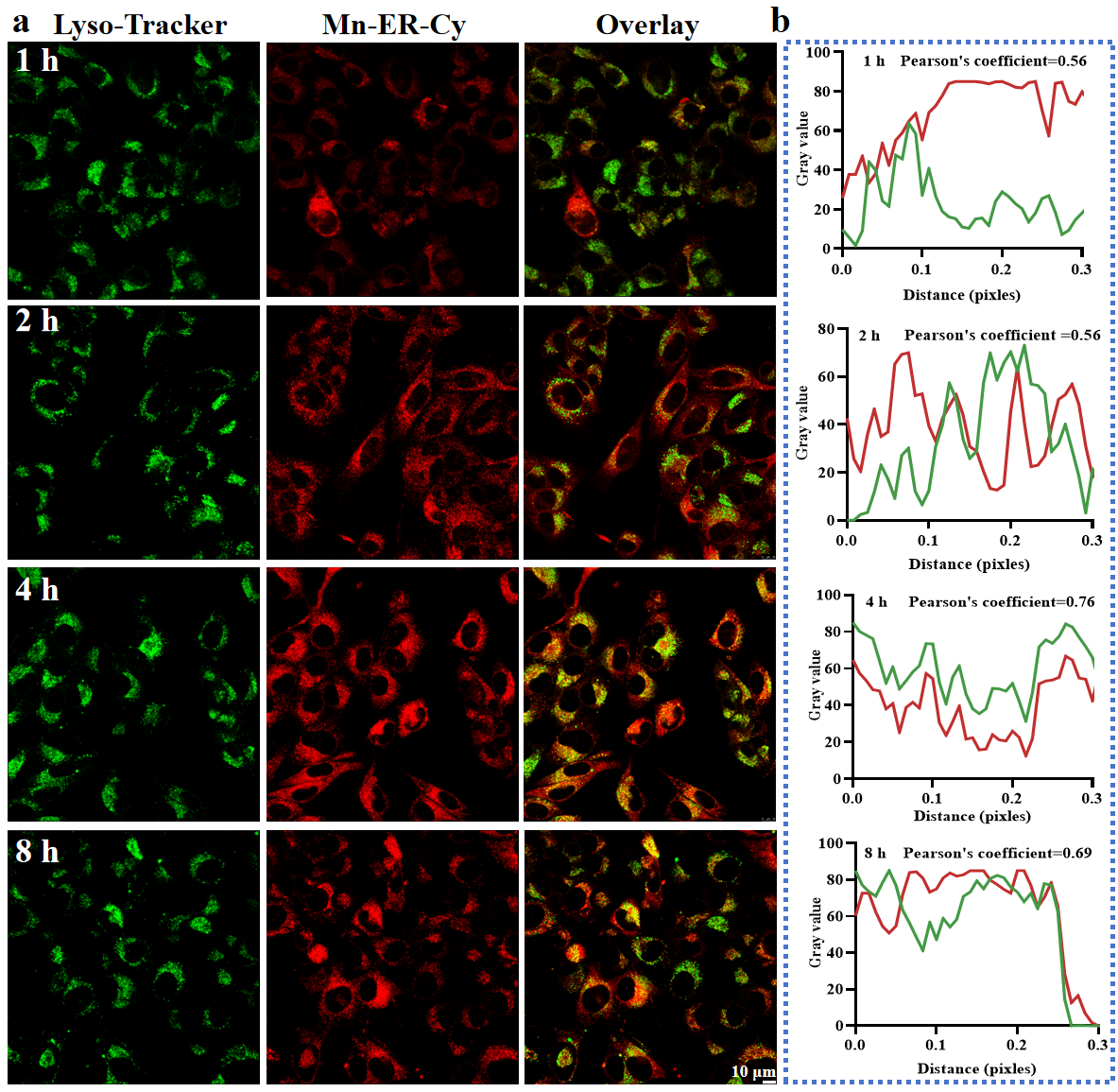 |
| --- |
| **Fig. S5** (**a**) CLSM images illustrating the subcellular co-localization of Mn-ER-Cy with Lysosome-Tracker Green in 4T-1 cells at various incubation time points. Scale bar = 10 µm. (**b**) Fluorescence topographic profiles of the co-localization of Mn-ER-Cy and Lysosome-Tracker Green at different time points. |

| 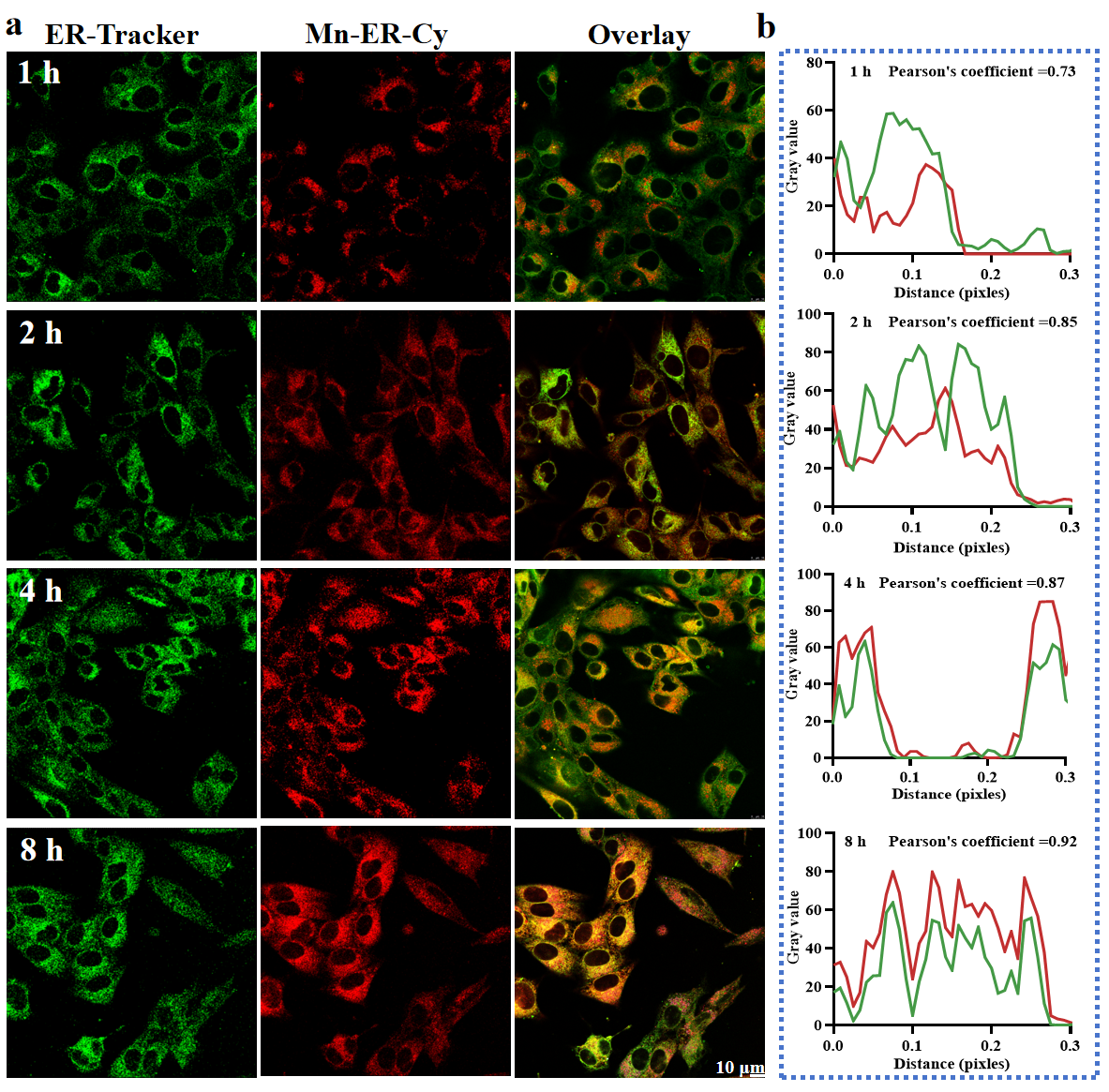 |
| --- |
| **Fig. S6** (**a**) CLSM images illustrating the subcellular co-localization of Mn-ER-Cy with ER-Tracker Green in 4T1 cells at various incubation time points. Scale bar = 10 µm. (**b**) Fluorescence topographic profiles of the co-localization of Mn-ER-Cy and ER-Tracker Green at different time points. |

|  |
| --- |
| **Fig. S7**(**a**-**b**) Heating and cooling curves of H_2_O and Mn-ER-Cy after exposure to 808-nm laser irradiation for 5 minutes, respectively. (**c**-**d**) Plot of time versus -ln(θ) for H_2_O and Mn-ER-Cy (where θ represents the driving force of temperature), based on the linear time data obtained during the cooling period, plotted against the negative natural logarithm of the driving force temperature, respectively. |

|  |
| --- |
| **Fig. S8** (**a**) Near-infrared fluorescence imaging of MnCO₃ and Mn-ER-Cy. (**b**) Singlet oxygen generation and (**c**–**d**) photothermal performance under 808 nm laser irradiation for 5 min. |

|  |
| --- |
| **Fig. S9** Flow cytometry analysis of the cytotoxicity of Mn-ER-Cy against different cells, including (**a**) mouse breast cancer 4T-1 cells, (**b**) human normal breast cells (MCF-10A), (**c**) human umbilical vein endothelial cells (HUVECs), and (**d**) type II alveolar epithelial cells (RLE-6TN). Statistical analyses were conducted using one-way ANOVA followed by Tukey’s test. *p < 0.05. |

|  |
| --- |
| **Fig. S10** (**a**) Live/dead staining, (**b**) ROS production, and (**c**) pyroptotic morphology of 4T-1 cells treated with MnCO₃ or Mn-ER-Cy under 808 nm laser irradiation for 5 min. White arrows indicate cell swelling characterized by large bubbles. |

|  |
| --- |
| **Fig. S11** CLSM images illustrating ROS levels in 4T-1 cells after various treatments for 24 hours. Scale bar = 100 µm. The results reveal the potential CDT and PDT effects of Mn-ER-Cy. Especially, a great number of ROS were detected after combined treatment of Mn-ER-Cy and laser irradiation (+). |

| 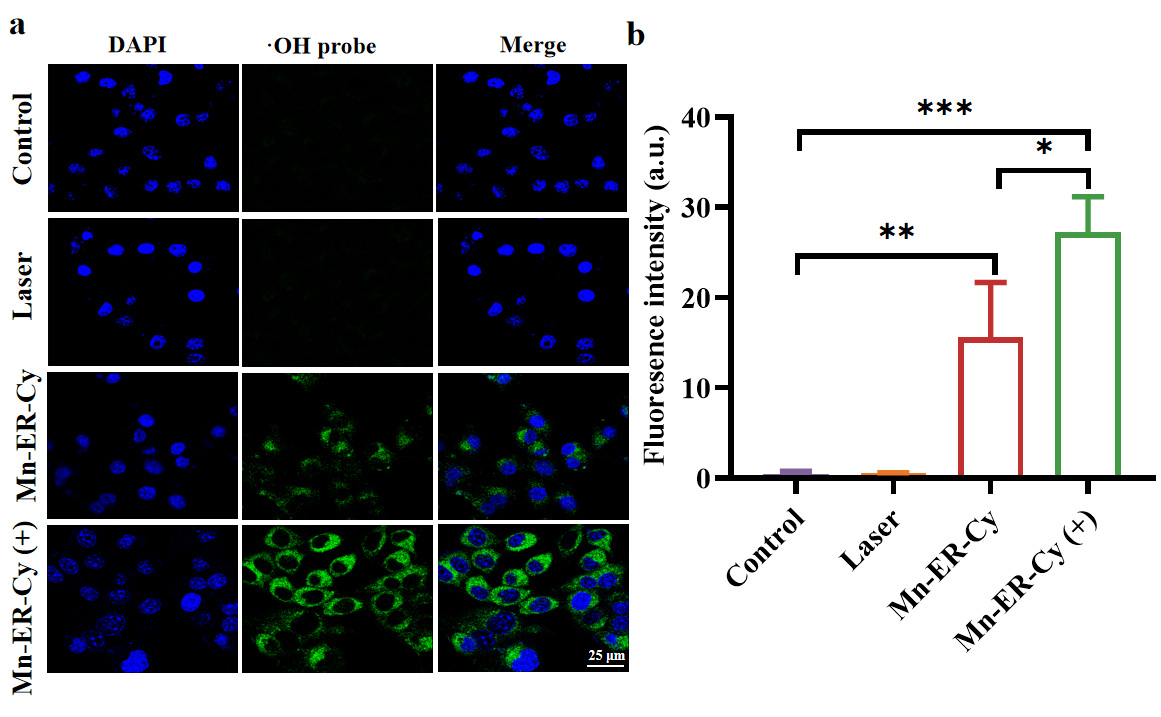 |
| --- |
| **Fig. S12** (**a**) CLSM images illustrating ·OH levels in 4T-1 cells after various treatments for 24 hours. Scale bar = 25 µm. (**b**) Semi-quantitative analysis of the fluorescence intensity as shown in panel a (n=3, mean ± s.d.). Statistical analyses were conducted using one-way ANOVA followed by Tukey’s test. *p < 0.05, **p < 0.01, ***p < 0.001. |
|  |

|  |
| --- |
| **Fig. S13** Fluorescence microscopy images illustrating the MMP in 4T-1 cells after various treatments for 24 hours. Red indicates normal MMP, while green indicates loss of MMP. Scale bar = 100 µm. |

|  |
| --- |
| **Fig. S14** (**a**) Cell scratch assay evaluating the migration ability of 4T1 cells after various treatments for 24 hours. Scale bar = 200 µm. (**b**) Quantitative analysis of the percentage of cell scratch wound healing in panel a (n=3, mean ± s.d.). Statistical analyses were conducted using one-way ANOVA followed by Tukey’s test. *p < 0.05, **p < 0.01. |

|  |
| --- |
| **Fig. S15** (**a**) Transwell invasion assay evaluating the invasion ability of 4T-1 cells after various treatments for 24 hours. Scale bar = 100 µm. (**b**) Semi-quantitative analysis of the number of cells that invaded the lower chamber in panel a (n=3, mean ± s.d.). Statistical analyses were conducted using one-way ANOVA followed by Tukey’s test. *p < 0.05. |

|  |
| --- |
| **Fig. S16** GSEA analysis the enrichment of unfold protein binding process in Mn-ER-Cy (+) treated cells. |

|  |
| --- |
| **Fig. S17** (**a**) CLSM images illustrating intracellular Ca^2+^ levels in 4T-1 cells after various treatments for 24 hours. Scale bar = 50 µm. (**b**) Semi-quantitative analysis of the fluorescence intensity as shown in panel a (n=3, mean ± s.d.). Statistical analyses were conducted using one-way ANOVA followed by Tukey’s test. **p < 0.01, ***p < 0.001. |

|  |
| --- |
| **Fig. S18** Inhibition of Mn-ER-Cy(+)-induced pyroptosis in 4T1 cells by the NLRP3-selective inhibitor MCC950. (**a**) Bright-field images showing 4T-1 cells morphology in each group. Black arrows indicate cell swelling characterized by large bubbles. (**b**) SEM images illustrating the morphological features of pyroptosis in each group. White arrows indicate membrane rupture and the presence of large pore-like structures. (**c**) Western blotting analysis of the relative protein levels of pyroptosis-related genes (NLRP3, GSDMD, GSDMD-N, and caspase-1) in each group.. |

|  |
| --- |
| **Fig. S19** Attenuation of Mn-ER-Cy(+)-induced pyroptosis in 4T1 cells by NLRP3 knockdown. (**a**-**b**) Validation of NLRP3 siRNA transfection efficiency by qRT-PCR and western blotting. (**c**) Bright-field images showing 4T-1 cells morphology in each group. Black arrows indicate cell swelling characterized by large bubbles. (**d**) SEM images illustrating the morphological features of pyroptosis in each group. White arrows indicate cell membrane rupture. (**e**) Western blotting analysis of the relative protein levels of pyroptosis-related genes (NLRP3, GSDMD, GSDMD-N, and caspase-1) in each group. |

|  |
| --- |
| **Fig. S20** (**a**) Photographs of cell culture supernatants from different treatment groups at 24 hours. (**b**-**c**) Quantitative analysis of pH values of cell culture supernatants from different groups at 4 hours and 12 hours (n=3, mean ± s.d.). Statistical analyses were conducted using one-way ANOVA followed by Tukey’s test. ***p < 0.001. |

|  |
| --- |
| **Fig. S21** (**a**) Infrared thermal images of tumor-bearing mice in PBS (+) group and Mn-ER-Cy (+) group. (**b**) Temperature change curve of the tumor site over time. |

|  |
| --- |
| **Fig. S22** (**a**-**b**) Photographs of tumors and corresponding weights on day 21 after various treatments (n=5, mean ± s.d.). (**c**-**d**) Tumor volume and body weight curves throughout the treatment period (n=5, mean ± s.d.). The experimental groups were categorized as follows: (I) PBS; (II) 808-nm laser (0.5 W/cm², 5 min); (III) Mn-ER-Cy; (IV) Mn-ER-Cy + 808-nm laser. Statistical analyses were conducted using one-way ANOVA followed by Tukey’s test. *p < 0.05, **p < 0.01, ***p < 0.001. |

| 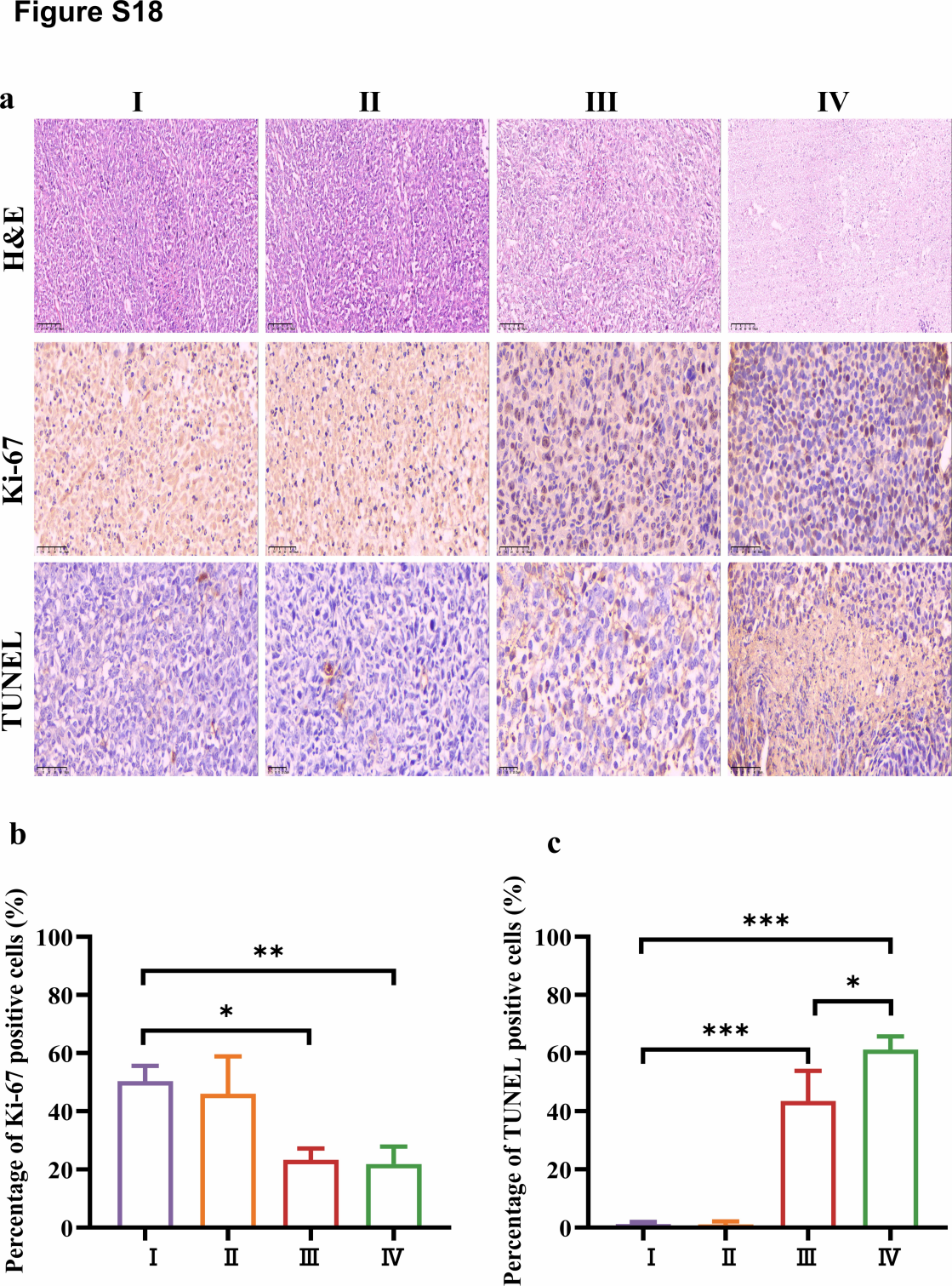 |
| --- |
| **Fig. S23** (**a**) H&E, Ki-67, and TUNEL staining of tumor sections on day 21 for each group. Scale bars = 100 µm, 50 µm, and 25 µm, respectively. (**b**-**c**) Semi-quantitative analysis of the percentage of Ki-67 and TUNEL positive cells in tumor tissues (n=3, mean ± s.d.). The experimental groups were categorized as follows: (I) PBS; (II) 808-nm laser (0.5 W/cm², 5 min); (III) Mn-ER-Cy; (IV) Mn-ER-Cy + 808-nm laser. Statistical analyses were conducted using one-way ANOVA followed by Tukey’s test. *p < 0.05, **p < 0.01, ***p < 0.001. |

|  |
| --- |
| **Fig. S24** (**a**-**b**) NIR fluorescence imaging of *ex vivo* tissues and blood samples at various time points. (**c**-**d**) Semi-quantitative analysis of the fluorescence intensity as shown in panels a and b. |

| 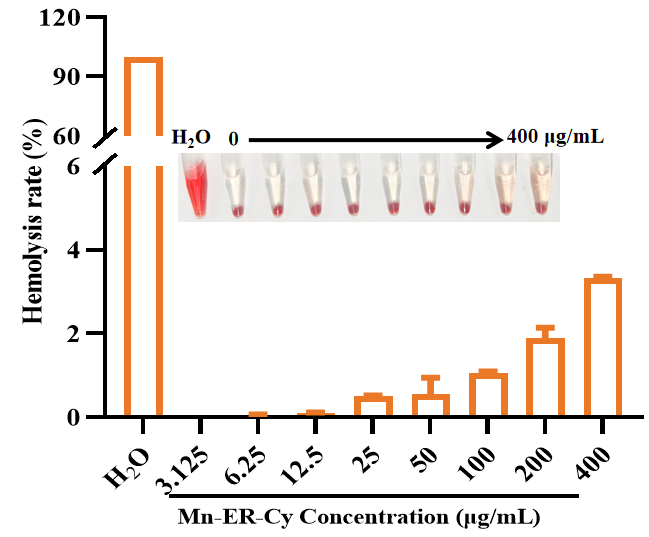 |
| --- |
| **Fig. S25** Photographs of the hemolysis test and the corresponding hemolysis percentage (n =3, mean ± s.d.). |

|  |
| --- |
| **Fig. S26** (**a**)-(**c**) Blood parameters for white blood cells (WBC), platelets (PLT), and red blood cells (RBC) of mice from each group on day 21 (n=3, mean ± s.d.). |

|  |
| --- |
| **Fig. S27** (**a-f**) Liver and kidney function parameters for alanine aminotransferase (ALT), aspartate aminotransferase (AST), urea, lactate dehydrogenase (LDH), creatinine (CREA), and creatine kinase (CK) in mice from each group on day 21 (n=3, mean ± s.d.). |

| 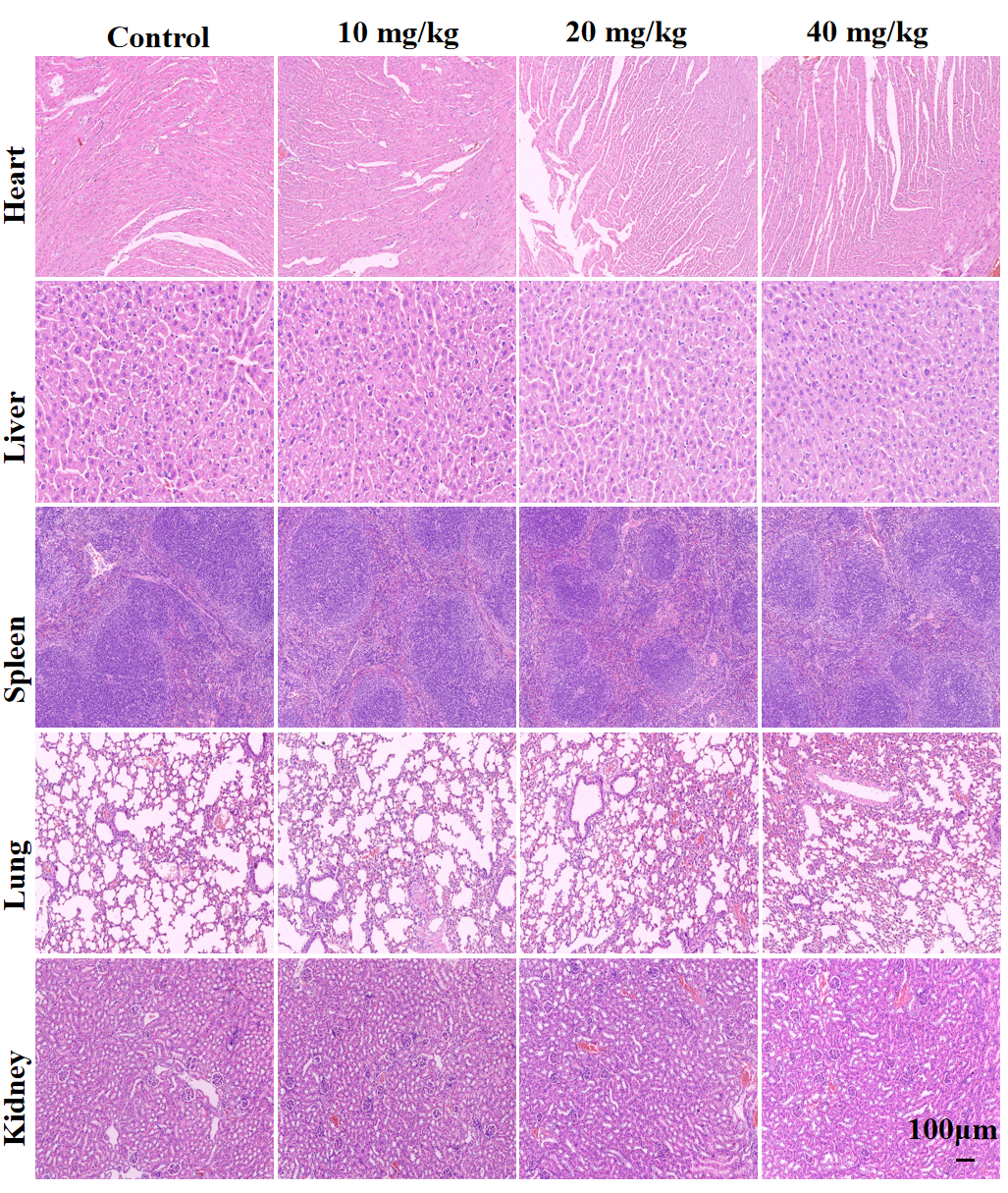 |
| --- |
| **Fig. S28** H&E staining images of the heart, liver, spleen, lungs, and kidneys from mice in each group on day 21. Scale bar = 100 µm. |

**Table S1. siRNA Sequences for Targeting NLRP3 Gene**

| **siRNA ID** | **Target Gene** | **Sense Strand Sequence (5'-3')** | **Anti-sense Strand Sequence (5'-3')** |
| --- | --- | --- | --- |
| siRNA-1 | NLRP3-Mus-1973 | CCAGGAGAGAACCUCUUAUTT | AUAAGAGGUUCUCUCCUGGTT |
| siRNA-2 | NLRP3-Mus-3088 | GCUGGAAUCUCUCCACAAUTT | AUUGUGGAGAGAUUCCAGCTT |
| siRNA-3 | NLRP3-Mus-2453 | GGACCUCAGUGACAAUACUTT | AGUAUUGUCACUGAGGUCCTT |
| siRNA-4 | NLRP3-Mus-2897 | CGGCCUUACUUCAAUCUGUTT | ACAGAUUGAAGUAAGGCCGTT |

**Table S1.** **Comparative Analysis of Mn-ER-Cy and Existing Mn-Based Nanocarriers**

| **Feature** | **Mn-based Nanocarriers**  **previously reported** | **Mn-ER-Cy**  **in this work** |
| --- | --- | --- |
| **Targeting Ability** | Passive tumor accumulation via the EPR effect or ligand modification (such as cRGD, mannose). Ref [1, 2] | Active targeting to tumor-cell ER organelles for precise therapy |
| **Subcellular Targeting** | Limited to general cellular uptake, with low subcellular specificity, A few studies target mitochondria or lysosomes.Ref [3, 4] | Selectively targets the endoplasmic reticulum (ER) of tumor cells. |
| **Photothermal Efficiency** | Typically below 50% efficiency.  Ref [5, 6] | High photothermal conversion efficiency of 51.4% under 808-nm irradiation |
| **Imaging Modalities** | Single-modal imaging (usually MRI or CT), or requires the combination of multiple materials to achieve multimodal imaging. Ref [7-9] | Dual-modal imaging: Near-infrared fluorescence (NIRF) and photoacoustic imaging. |
| **Antitumor Mechanism** | Limited to a single therapeutic modality (chemotherapy, PTT, etc.), or requires the combination with chemotherapeutic drugs for synergistic effects. Ref [10, 11] | Multifunctional therapy combining PTT, PDT, and Mn²⁺-mediated CDT. |

**Supplementary Materials and Methods**

**Material characterization**

The morphology of the synthesized Nanocubes (NCs) was characterized using transmission electron microscopy (TEM, JEOL JEM-F200, Japan) and high-angle annular dark field scanning transmission electron microscopy (HAADF-STEM, JEOL JEM 2100F). Fourier transform infrared spectroscopy (FTIR, Thermo Fisher Scientific Nicolet iS5, USA) was employed for the qualitative and quantitative analysis of functional groups in ER-Cy, MnCO₃, and Mn-ER-Cy. X-ray powder diffraction (XRD, Rigaku Ultima IV, Japan) was used to determine the composition by examining the spatial distribution patterns and intensities of diffraction lines in these materials. X-ray photoelectron spectroscopy (XPS, Thermo Scientific K-Alpha, USA) provided a semi-quantitative analysis of elemental composition and chemical states. Additionally, energy-dispersive spectroscopy (EDS, Oxford X80T, UK) assessed elemental concentration and distribution. A UV-visible spectrophotometer (UV-3600, Shimadzu, Japan) and a fluorescence spectrophotometer (Thermo Fisher, USA) were utilized to analyze the UV-Vis-NIR absorption and fluorescence emission spectra of Mn-ER-Cy.

**Dynamic light scattering method (DLS) and zeta potential**

MnCO_3_ and Mn-ER-Cy were dispersed in deionized water at a concentration of 50 μg/mL, and sonicate for 5 minutes to achieve thorough dispersion. Then, the hydrated particle size and zeta potential of the nanoparticles were detected using the Malvern Nano-ZS90 dynamic light scattering particle size analyzer.

**Cell viability assay**

4T-1 cells were seeded at a density of 5000 cells per well into a 96-well plate and incubated with different concentrations (0, 12.5, 25, 50, 100, 200 μg/mL) of Mn-ER-Cy for 24 hours. Additionally, to evaluate the phototoxicity of Mn-ER-Cy, the cells were co-incubated with the drug for 4 hours followed by exposure to 808-nm NIR laser irradiation (0.8 W/cm²) for 5 minutes, and then continued to culture for another 24 hours. Cell viability was assessed using CCK-8 reagent (HY-K0301, MedChem Express) according to the manufacturer's instructions. The relative cell viability was calculated using the following formula: Relative cell viability (%) = (OD_sample - OD_blank) / (OD_control - OD_blank) × 100%.

**Live and dead assay**

4T-1 cells were seeded at a density of 3×10^5 cells per well into a 6-well plate. After 24 hours of treatment, the old medium was removed, and the wells were washed three times with PBS. Subsequently, 1 mL of Calcein-AM/Propidium iodide (C542, Dojindo) working solution was added to each well, and incubation was continued at 37 °C for 1 hour. Cells were then observed under a fluorescence microscope for viability, with live cells appearing green and dead cells appearing red. (Calcein-AM_Ex/Em_=494 nm/517 nm, PI_Ex/Em_=535 nm/617 nm.)

**Colony formation assay**

4T-1 cells were seeded into a 6-well plate and treated according to the experimental groups for 24 hours. Subsequently, the cells were digested with trypsin and re-seeded into a new 6-well plate at a density of 1,000 cells per well. The cells were cultured under standard conditions, and the culture medium was replaced every three days. Once the control group exhibited distinguishable colonies, the cells were fixed with 4% paraformaldehyde for 30 minutes and then stained with crystal violet for 20 minutes. After staining, the cells were washed with running water for 5 minutes and allowed to dry. Finally, images of the colonies were captured, and colony counts were conducted using ImageJ software, defining colonies as clusters of more than 50 cells.

**Mitochondrial membrane potential assay**

4T-1 cells were seeded in a 6-well plate at a density of 2 × 10^5 cells per well and subjected to the designated treatments. Afterward, 1 mL of JC-1 working solution, diluted 1:200, was added and incubated for 20 minutes at 37 °C. Following incubation, the cells were washed twice with 1X JC-1 staining buffer, after which 1 mL of cell culture medium was added. Changes in mitochondrial membrane potential were then observed using a fluorescence microscope (JC-1 monomers Ex/Em = 514 nm/529 nm; JC-1 aggregates Ex/Em = 585 nm/590 nm).

**Wound scratch assay**

Following the designated treatments, 4T-1 cells were seeded in a 6-well plate. When the cells reached 90% confluence, vertical scratches were created on the cell surface using a sterile pipette tip. The cells were then washed three times with PBS to remove any detached cells. Subsequently, 1 mL of complete culture medium was added, and photographs were captured under an inverted microscope to document the initial scratch position and width (0 hours). After a 24-hour incubation period, a second set of images of the cell scratches was obtained. Scratch areas at both 0 hours and 24 hours were analyzed using ImageJ software to calculate the scratch healing rate for each experimental group.

**Cell invasion assay**

Following the designated treatments, 4T-1 cells were resuspended in serum-free DMEM at a concentration of 2 × 10^5 cells/mL. A total of 300 μL of the suspension was added to the upper chamber of Matrigel-coated Transwell plates, with 600 μL of DMEM containing 10% FBS in the lower chamber. The plates were incubated in a cell culture incubator for 24 hours. After incubation, the chambers were fixed in 4% paraformaldehyde at room temperature for 10 minutes. Subsequently, 1 mL of 0.5% crystal violet was added for 20 minutes of staining, followed by three washes with PBS to remove excess dye. After air drying, images were captured under a microscope, and the number of invasive cells was quantified using ImageJ software.

**Immunofluorescence staining assay**

4T-1 cells were seeded in a 35 mm culture dish and treated according to the designated experimental groups, followed by an additional 24-hour incubation period. The cells were subsequently fixed with 4% paraformaldehyde for 15 minutes and permeabilized at room temperature using 0.3% Triton X-100 for 10 minutes. An antibody blocking solution was then added, and the samples were incubated at room temperature for 2 hours. Following blocking, primary antibodies against CHOP (cat#: YM3668, ImmunoWay), GRP78 (cat#: YM3508, ImmunoWay), Calreticulin (cat#: ab92516, Abcam), and HMGB1 (cat#: ab18256, Abcam) were applied, and the samples were incubated overnight at 4 °C. Following this, a secondary antibody working solution of goat anti-rabbit IgG H&L (FITC) diluted at 1:500 was added and incubated in the dark at room temperature for 2 hours. The nuclei were stained with DAPI for 15 minutes, and finally, 1 mL of anti-fade mounting medium was added. The samples were then observed and imaged using CLSM.

**Cell morphology observation**

4T-1 cells were seeded in a 6-well plate and treated according to the designated experimental groups, followed by a 24-hour incubation period. For bright field observation, the cell morphology was directly examined using an inverted microscope. For transmission electron microscopy (TEM) observation, 1.5 mL of glutaraldehyde fixative was added, and the samples were pre-fixed at room temperature for 20 minutes. The cells were then gently scraped with a specialized scraper and transferred to a 1.5 mL Eppendorf tube, followed by centrifugation at 1000 rpm for 5 minutes to pellet the cells. The supernatant was discarded, and the cell pellet was resuspended in 1 mL of 2.5% glutaraldehyde fixative at 4 °C for overnight fixation. After fixation, the samples were rinsed three times with 0.1 M phosphate buffer (pH 7.0), with each rinse lasting 15 minutes. The samples were then fixed with 1% osmium tetroxide for 2 hours and dehydrated using a gradient of ethanol (30%, 50%, 70%, 80%, 90%, 100%) for 15 minutes at each concentration, followed by 20 minutes in pure acetone. Next, the samples were immersed in a mixture of acetone and resin (3:1 for 2 hours; 1:1 for 3 hours; 1:3 for 3 hours), and then placed in pure resin overnight. The infiltrated samples were transferred into molds and subjected to gradient heating at 35 °C, 60 °C, and 80 °C, with each step lasting 5 hours for embedding. After coarse trimming, ultra-thin sections (70–90 nm) were obtained using a LEICA EM UC7 ultramicrotome. The sections were stained with uranyl acetate for 15 minutes and lead citrate for 5 minutes, then allowed to dry prior to observation under a transmission electron microscope (FEI TECNAI G2 12).

For scanning electron microscopy (SEM) observation, 4T-1 cells were seeded onto 18 mm cover slips and treated according to the experimental groups, followed by a 24-hour incubation. After incubation, pre-cooled 2.5% glutaraldehyde fixative at 4 °C was added, and the samples were stored in a refrigerator overnight. The samples were subsequently dehydrated using a gradient of ethanol concentrations (30%, 50%, 70%, 80%, 90%, 95%, 100%), with each concentration processed twice for 15 minutes. Liquid carbon dioxide was then introduced to replace the ethanol for 30 minutes. Following this, a conductive treatment was conducted using a vacuum sputter coater, positioning the samples 10 to 15 cm from the evaporation source and allowing them to rotate during gold sputtering at 10 kV for 1000 seconds. Finally, the samples were examined using a scanning electron microscope (SEM, Hitachi SU8020).

***In vitro* BMDCs maturation detection**

Bone marrow-derived dendritic cells (BMDCs) were isolated from six-week-old female BALB/c mice using previously reported methods [12]. Following isolation, 25 ng/mL recombinant mouse GM-CSF (cat#:315-03, PeproTech) and 10 ng/mL IL-4 (cat#:214-14, PeproTech) were used to stimulate the BMDCs, which were cultured at 37 °C in a 5% CO2 atmosphere. On day 7 of culture, the medium was gently agitated to collect both suspended and adherent cells that had fully differentiated into dendritic cells. Subsequently, the BMDCs were co-cultured with supernatants from four treatment groups of 4T-1 cells for 24 hours. After co-culture, the cells were stained with anti-CD11c (Cat#:12-0114-82, eBioscience), anti-CD80 (Cat#:17-0801-82, eBioscience), and anti-CD86 (Cat#:25-0862-82, eBioscience) antibodies, and the maturation of each BMDC group was analyzed using flow cytometry.

**Inflammatory cytokine detection**

To detect inflammatory cytokines for *in vitro* experiments, 4T-1 cells were seeded at a concentration of 1 × 10^5 cells/mL in a 6-well plate and treated according to the specified experimental groups. After a 24-hour treatment period, the levels of IL-1β (cat#:EK0394, Boster) and IL-18 (cat#:EK0433, Boster) in the cell supernatant were quantified following the manufacturer’s instructions.

To assess cytokine secretion in the blood of mice, 4T-1 tumor cells were inoculated subcutaneously into the right hind limb of female BALB/c mice. Once the tumor volume reached 100 mm³, the mice were randomly divided into four groups: Control group, Laser group, Mn-ER-Cy group, and Mn-ER-Cy (+) (n=3 per group). On the 7th day post-treatment, peripheral blood was collected from the mice using the eye exsanguination method, and serum was separated. Subsequently, the levels of IL-1β and IL-18 were evaluated according to the manufacturer’s instructions.

**Western blotting analysis**

4T-1 cells were cultured in a 6-well plate for 24 hours and then subjected to group-specific treatments for an additional 24 hours, as previously described. After removing the old culture medium, RIPA lysis buffer containing protease and phosphatase inhibitors was added for protein extraction. Protein concentrations from each group were determined using a BCA assay kit according to the manufacturer's instructions (Cat#:P0010, Beyotime). The extracted proteins were boiled at 100 °C for 5 minutes, and appropriate volumes based on protein concentrations were loaded onto SDS-PAGE for electrophoresis. Proteins were subsequently transferred onto a polyvinylidene fluoride (PVDF) membrane, which was blocked with non-fat milk at room temperature for 1 hour. The membrane was then incubated overnight at 4 °C with primary antibodies against NLRP3 (Cat#:68102-1-Ig, Proteintech), caspase 1 (Cat#:22915-1-AP, Proteintech), and GSDMD-N terminal (Cat#:B7001, ImmunoWay). β-actin (700608, ZEN Bio) served as a loading control. After that, the membrane was incubated with secondary antibodies at room temperature for 1 hour. Protein bands were visualized using an enhanced chemiluminescence detection reagent (Cat#:P0018FM, Beyotime), and images were analyzed semi-quantitatively using ImageJ software.

***In vitro* hemolysis assay**

Blood samples were collected from BALB/c mice via orbital puncture into anticoagulant tubes containing EDTA-Na_2_. The samples were then centrifuged at 1000 g for 10 minutes, and this process was repeated three times to effectively remove collagen fibers. Following centrifugation, 100 μL of the processed blood was aliquoted into Eppendorf tubes, and 900 μL of either deionized water (positive control), PBS (pH 7.4, negative control), or varying concentrations of Mn-ER-Cy (3.125, 6.25, 12.5, 25, 50, 100, 200, and 400 μg/mL) was added to each tube. The mixtures were thoroughly mixed and incubated at 37 °C in a shaking incubator for 2 hours. After incubation, the samples were centrifuged again at 1000 g for 10 minutes. Eppendorf tubes were photographed to visually document any hemolysis. Subsequently, 200 μL of the supernatant was transferred to a 96-well plate, and the absorbance of these supernatants was measured at 540 nm using a multifunctional microplate reader. The hemolysis rate was calculated using the formula: The hemolysis rate was calculated using the formula: Hemolysis Rate (%) = [(OD_sample − OD_negative) / (OD_positive − OD_negative)] × 100 %.

**References**

1. Zhang Y, Yu X, Luo L, Xu Y, Zhang H, Mao Z et al. Engineered manganese-BODIPY coordinated nanoadjuvants for enhanced NIR-II photo-metalloimmunotherapy. J Control Release. 2024;376:1115-1129.

2. Peng D, Cheng L, Tang J, Liu Z, Xue Y, Liu J. Engineered NK Exosomes Captured Antigens In Situ for Enhanced Tumor Immunotherapy. ACS Appl Mater Interfaces. 2025;17(16):23740-23752.

3. Li S, Chen Y, He P, Ma Y, Cai Y, Hou X et al. Aggregation-Induced Emission (AIE) Photosensitizer Combined Polydopamine Nanomaterials for Organelle-Targeting Photodynamic and Photothermal Therapy by the Recognition of Sialic Acid. Adv Healthc Mater. 2022;11(15):e2200242.

4. Liu Z, Tang Z, Yin Y, Wan M, Zhan J, Ren L. A Microneedle Patch Delivers Mitochondria- and Lysosomes- Dual Targeting Prodrug-Like Photosensitizers with Regulated Photoactivity for Precise Photodynamic Therapy. Adv Healthc Mater. 2025;14(9):e2403954.

5. Shen Y, Li X, Huang H, Lan Y, Gan L, Huang J. Embedding Mn(2+) in polymer coating on rod-like cellulose nanocrystal to integrate MRI and photothermal function. Carbohydr Polym. 2022;297:120061.

6. Zhao R, Zhu Y, Zhou J, Liu B, Du Y, Gai S et al. Dual Glutathione Depletion Enhanced Enzyme Catalytic Activity for Hyperthermia Assisted Tumor Therapy on Semi-Metallic VSe(2)/Mn-CS. ACS Nano. 2022;16(7):10904-10917.

7. Huang P, Tang Q, Li M, Yang Q, Zhang Y, Lei L et al. Manganese-derived biomaterials for tumor diagnosis and therapy. J Nanobiotechnology. 2024;22(1):335.

8. Zhu B, Zhang M, Chen Q, Li Z, Chen S, Zhu J. Starvation-assisted and photothermal-thriving combined chemo/chemodynamic cancer therapy with PT/MR bimodal imaging. Biomater Sci. 2023;11(6):2129-2138.

9. Xie X, Liu W, Zhu W, Zhang G, Dai Y, Wu J et al. A cell penetrating peptide-modified magnetic/fluorescent probe for in vivo tracking of mesenchymal stem cells. J Biomed Mater Res A. 2022;110(12):1881-1891.

10. Liu Z, Deng X, Wang Z, Guo Y, Hameed MMA, El-Newehy M et al. A biomimetic therapeutic nanovaccine based on dendrimer-drug conjugates coated with metal-phenolic networks for combination therapy of nasopharyngeal carcinoma: an in vitro investigation. J Mater Chem B. 2025.

11. Heng C, Liu W, Zheng X, Ma X, Hui J, Fan D. Dopamine and DNA functionalized manganese whitlockite nanocrystals for magnetic resonance imaging and chemo-photothermal therapy of tumors. Colloids Surf B Biointerfaces. 2023;222:113120.

12. Luo S, Luo X, Wang X, Li L, Liu H, Mo B et al. Tailoring Multifunctional Small Molecular Photosensitizers to In Vivo Self-Assemble with Albumin to Boost Tumor-Preferential Accumulation, NIR Imaging, and Photodynamic/Photothermal/Immunotherapy. Small. 2022;18(27):e2201298.
